# Emerging quantum critical phase in a cluster spin-glass

**DOI:** 10.1093/nsr/nwaf483

**Published:** 2025-11-06

**Authors:** Fang Zhang, Tao Feng, Yurong Ruan, Xiaoyuan Ye, Bing Wen, Liang Zhou, Minglin He, Zhaotong Zhuang, Liusuo Wu, Hongtao He, Peijie Sun, Zhiyang Yu, Weishu Liu, Wenqing Zhang

**Affiliations:** Department of Materials Science and Engineering, Southern University of Science and Technology, Shenzhen 518055, China; Department of Physics, University of California at Berkeley, Berkeley 94720, USA; Department of Materials Science and Engineering, Southern University of Science and Technology, Shenzhen 518055, China; Department of Materials Science and Engineering, Southern University of Science and Technology, Shenzhen 518055, China; State Key Laboratory of Photocatalysis on Energy and Environment, College of Chemistry, Fuzhou University, Fuzhou 350108, China; Department of Materials Science and Engineering, Southern University of Science and Technology, Shenzhen 518055, China; Department of Physics, The University of Hong Kong, Hong Kong 999077, China; Department of Physics, Southern University of Science and Technology, Shenzhen 518055, China; Department of Mechanical and Energy Engineering, Southern University of Science and Technology, Shenzhen 518055, China; Beijing National Laboratory for Condensed Matter Physics, Institute of Physics, Chinese Academy of Sciences, Beijing 100190, China; Department of Physics, Southern University of Science and Technology, Shenzhen 518055, China; Department of Physics, Southern University of Science and Technology, Shenzhen 518055, China; Beijing National Laboratory for Condensed Matter Physics, Institute of Physics, Chinese Academy of Sciences, Beijing 100190, China; State Key Laboratory of Photocatalysis on Energy and Environment, College of Chemistry, Fuzhou University, Fuzhou 350108, China; Department of Materials Science and Engineering, Southern University of Science and Technology, Shenzhen 518055, China; Department of Materials Science and Engineering, Southern University of Science and Technology, Shenzhen 518055, China; Shenzhen Institute for Quantum Science and Engineering, Southern University of Science and Technology, Shenzhen 518055, China; Shenzhen Municipal Key-Lab for Advanced Quantum Materials and Devices & Guangdong Provincial Key Lab for Computational Science and Materials Design, Southern University of Science and Technology, Shenzhen 518055, China

**Keywords:** quantum phase transition, heavy-fermion metal, cluster spin-glass, random magnetic moments

## Abstract

The study of strong electron correlations has significantly advanced the frontiers of condensed matter physics, especially in relation to correlation-driven quantum phase transitions (QPTs). In the vicinity of QPTs, quantum critical fluctuations of multiple degrees of freedom enable the emergence of exotic many-body states and quantum critical behaviours beyond the Landau paradigm. Recently, magnetic frustration, traditionally associated with insulating magnets, has been recognized as pivotal to investigating new phases of matter in correlation-driven Kondo breakdown QPTs that are not clearly associated with broken symmetry. The nature of these new phases, however, remains underexplored. Here, we report quantum criticalities emerging from a cluster spin-glass in the heavy-fermion metal TiFe_x_Cu_2x__−__1_Sb, where frustration originates from intrinsic disorder. Specific heat and magnetic Grüneisen parameter measurements under varying magnetic fields exhibit quantum critical scaling, indicating a quantum critical point (QCP) near 0.13 Tesla. As the magnetic field increases, the cluster spin-glass phase is progressively suppressed. Upon crossing the QCP, resistivity and Hall effect measurements reveal enhanced screening of local moments and an expanding Fermi surface, consistent with the Kondo breakdown scenario. Our findings uncover a new family of iron-based heavy-fermion metals with intricate interplay of multiple degrees of freedom, enabling the exploration of unconventional excitations and exotic quantum critical states and behaviours.

## Teaser

Local quantum phase transition is observed in an off-stoichiometric Heusler-like magnet with spin-glass behaviour, essentially confirming that Kondo-breakdown quantum criticality can occur in magnetically disordered systems without long-range magnetic interaction.

## INTRODUCTION

Magnetic frustration in antiferromagnetic (AFM) insulators can induce quantum spin fluctuations that suppress long-range magnetic order (LRMO), leading to highly spin-correlated ground states found in exotic materials such as quantum spin-liquids and spin-glasses [1,2]. Magnetic frustration can also occur in heavy-fermion metals, where it interacts with the Ruderman–Kittel–Kasuya–Yosida (RKKY) magnetic interactions among local moments, as well as their magnetic screening by conduction electrons, a phenomenon known as the Kondo effect [3]. When the interplay between these competing forces and associated quantum fluctuations becomes prominent, it can lead to novel quantum critical phases and behaviours. By exploring different types of quantum phase transitions (QPTs) and their evolution, we can gain unprecedented insights into the origins of strange-metal quantum criticality, one of the most pressing questions in many-body physics [4].

Recent studies on QPTs, particularly those transitions from a long-range AFM or ferromagnetic (FM) order to a paramagnetic heavy-fermion liquid, have advanced modern theories of quantum criticality [5–7]. This emerging framework emphasizes that the behaviour of local moments is as critical as the long-wavelength fluctuations of the order parameter in the canonical Landau paradigm [8,9]. A crucial corollary of this theory, known as the Kondo breakdown scenario, is that QPTs can arise from the transition of spin-glasses to a heavy fermion-liquid with an enlarged Fermi surface volume [10]. Extensive theoretical investigations have pointed out that magnetically frustrated systems, featuring both RKKY and Kondo interactions, may undergo such QPTs through varying non-thermal control parameters, such as magnetic field, pressure or atomic substitution [11–16].

LiV_2_O_4_ is an early candidate proposed to host these intriguing QPTs, exhibiting magnetic frustration due to the varied valences of two vanadium atoms. Despite the potential logarithmic temperature dependence of its specific heat [17], a signature of the onset of the quantum critical phase [18], and the observation of a spin-glass state upon Zn-doping [19], subsequent studies have shown that the Kondo effect in LiV_2_O_4_ is negligible. Instead, its heavy-fermion behaviours primarily arise from the lightly doped Mott insulator nature of the strongly correlated a_1_*_g_* band [20]. While other materials, highlighted by Pr_2_Ir_2_O_7_ [21], Ge-substituted YbRh_2_Si_2_ [22] and CePdAl [23], have been investigated as potential candidates, conclusive experimental evidence linking spin-correlated quantum states to QPTs remains elusive, particularly in relation to a spin-glass state.

In this study, we report a new family of iron-based heavy-fermion metals, TiFe_x_Cu_2x−1_Sb, exemplified by *x* = 0.70, where magnetic frustration originates from inherent disorder within the Fe and Cu sites. Through detailed ultra-low temperature thermodynamic measurements, including specific heat and magnetic Grüneisen parameter, we observe a magnetic field-induced quantum critical point (QCP). Upon crossing this QCP, Hall measurements show an increase in the Fermi surface volume consistent with the Kondo breakdown scenario, as partially supported by resistivity measurements that suggest enhanced screening of local moments. Additionally, direct current (dc) and alternating current (ac) magnetic susceptibility measurements identify a cluster spin-glass ground state where magnetic entities are spin clusters, not isolated atomic spins, which are progressively suppressed with increasing magnetic field.

## RESULTS AND DISCUSSION

### Crystal structure and disorder-induced local moments

**Figure 1.**
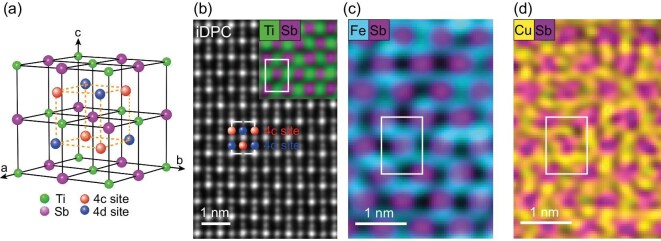
Crystal structure of TiFe_0.7_Cu_0.4_Sb. (a) Crystal structure of off-stoichiometry TiFe_0.7_Cu_0.4_Sb. Ti and Sb atoms form two interpenetrating face-centered cubic lattices, while Fe and Cu atoms partially and randomly occupy the 4c (red) and 4d (blue) sites within the structure. (b) iDPC image along the [110] direction. The white rectangle outlines a conventional unit cell; red and blue circles mark the positions of the 4c and 4d sites, respectively. Inset, EDS maps for Ti and Sb atoms, illustrating their periodic distribution and regular structural order. (c and d) EDS maps for Fe atoms (c) and for Cu atoms (d), both highlighting their random distribution and indicating chemical disorder when occupying the 4c and 4d sites.

Figure 1a shows the schematic crystal structure of TiFe_0.7_Cu_0.4_Sb (see Section S1, Fig. S1), an off-stoichiometric compound with Heusler-like characteristics [24] that belongs to the $F\bar{4}3m$ (No. 216) space group. In this structure, Ti and Sb atoms form the skeleton of a face-centered cubic lattice, while Fe and Cu atoms are expected to partially occupy both the 4c and 4d Wyckoff sites in a disordered manner [25]. The uniform distribution and expected atomic arrangements of the atoms are confirmed through the integrated differential phase contrast (iDPC) imaging, depicted in Fig. 1b (see Section S2, Fig. S2). Specifically, energy-dispersive spectroscopy (EDS) maps for Fe (Fig. 1c) and Cu (Fig. 1d) reveal their random spatial distribution, indicating significant chemical disorder between these two competing elements across the 4c and 4d sites. In contrast, neighboring Ti and Sb atoms exhibit periodic arrangements (as shown in the inset of Fig. 1b).

This unique crystal structure of TiFe_0.7_Cu_0.4_Sb leads to randomly distributed local moments, primarily originating from the partially filled *e_g_* orbitals of Fe atoms. Due to disorder-induced variations in the crystal field environment around Fe atoms at different sites, their magnetic moments show considerable variation, ranging from 0.0 to 1.5 *μ_B_*, as demonstrated by our ab initio calculations (see Section S3, Fig. S3). This behaviour can be attributed to Fe being generally non-magnetic in a half-Heusler structural environment or weakly magnetic in a full-Heusler environment, both of which can coexist in such a disordered system. Low-temperature magnetization measurements, *M*(*μ_0_H*), reveal a saturation moment of approximately 0.05 *μ_B_* per Fe atom (see Section S4, Figs S4 and S5), suggesting that most Fe atoms possess negligible magnetic moments. This combination of structural disorder and small average magnetic moments identifies TiFe_0.7_Cu_0.4_Sb as a dilute Kondo disordered system [26], characterized by magnetic ions randomly dispersed throughout the lattice.

### Thermodynamic evidence for a magnetic field-induced QCP

**Figure 2.**
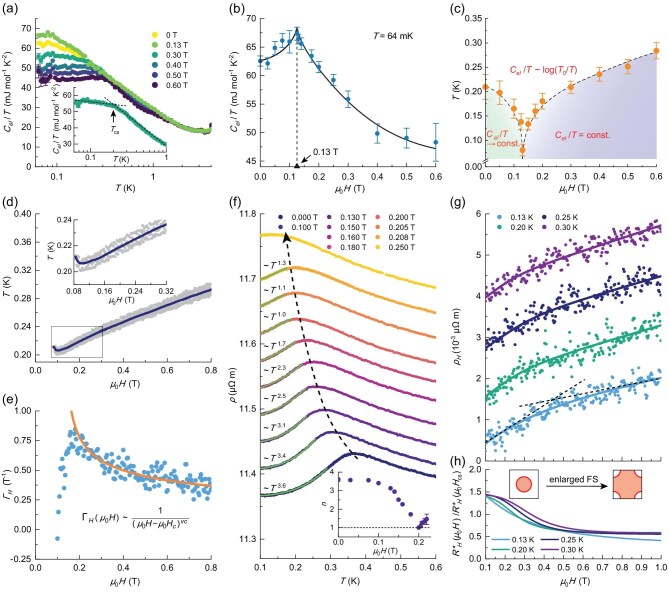
Thermodynamic and transport measurements of TiFe_0.7_Cu_0.4_Sb. (a) Electronic specific heat coefficient, *C_el_*(*T*)/*T*, under various magnetic fields. Errors at low temperatures are due to nuclear Schottky contributions to specific heat. Inset: *C_el_*(*T*)/*T* at *μ_0_H* = 0.30 T. The arrow indicates the temperature scale *T_cs_* (see main text). (b) *C_el_*(*T*)/*T* measured at *T* = 64 mK, which shows a pronounced peak near *μ_0_H* ∼ 0.13 T. The black solid line serves as a visual guide. (c) The temperature scale *T_cs_* at various magnetic fields. Different regimes demonstrate different scaling behaviours of *C_el_*(*T*)/*T*, as highlighted in red text. (d) Magnetocaloric effect measured with varying magnetic fields down to *μ_0_H* = 0.08 T. Below this field, the data become unreliable due to noise from magnetic flux transitions. Inset shows zoom in plot between *μ_0_H* = 0.08 and 0.32 T. (e) Magnetic Grüneisen parameter, *Γ_H_* = *dT*/*Td* (*μ_0_H*), as a function of the magnetic field *μ_0_H*. The sold line is the best fit of data, with *μ_0_H_c_* = 0.13 T (see main text). (f) Resistivity *ρ*(*T*) under various magnetic fields. Datapoints are vertically shifted for better comparison. The low-temperature behaviour of resistivity is fitted to *ρ*(*T*) ∝ *T^n^*, with the exponent *n* plotted in the inset. The dashed black curve with an arrow indicates the shift in the maximum of *ρ*(*T*). (g) Magnetic field dependence of the Hall resistivity, *ρ_H_* (*μ_0_H*), at different temperatures. Datapoints are vertically shifted for better comparison. Solid lines show the best fit, $\smallint R_H^*( {{\mu }_0H} )dH$, to the data. Black dashed lines serve as visual guides. (h) Effective Hall coefficient, $R_H^*$(${\mu }_0H$), at various temperatures. As *μ_0_H* increases, $R_H^*$(${\mu }_0H$) decreases from large to small values, indicating a transition from a small to a larger Fermi surface (FS) volume within the single-carrier model.

We conducted specific heat measurements on single-phase polycrystalline TiFe_0.7_Cu_0.4_Sb samples, with results shown in Fig. 2a–c. To isolate the electronic specific heat coefficient, *C_el_*(*T*), we analyzed the total specific heat, *C_tot_*(*T*), which consists of contributions from nuclear (*C_nuc_*), magnetic Schottky (*C_sch_*), phonon (*C_ph_)* and electronic (*C_el_*) components (see Sections S5–7, Figs S6–S8). Figure 2a shows that *C_el_*(*T*)/*T* transitions from a log (1/*T*)-dependent behaviour at high temperatures to either a constant value (at high fields) or an approach toward a constant value (at low fields) upon cooling. This transition is characterized by a crossover temperature scale *T_cs_* (see Section S8, Fig. S9), which decreases with the applied fields for *μ_0_H* < *μ_0_H_c_*, but increases for *μ_0_H* > *μ_0_H_c_*, where the critical field is approximately *μ_0_H_c_* ∼ 0.13 T (Fig. 2c). As a result, a fan-shaped critical regime emerges, in which the electronic specific heat, *C_el_*(*T*)/*T*, follows a logarithmic dependence on temperature. The power-law divergence of *C_el_*(*T*) at *μ_0_H_c_* ∼ 0.13 T exhibits a logarithmic dependence, *C_el_*(*T*)/*T* ∝ log (*T*_0_/*T*), over a broad temperature range (0.08–2 K), with *T*_0_ = 7.68(1) K being the characteristic temperature associated with spin fluctuation energies. This feature, as observed over an extended temperature range, is widely regarded as a hallmark of quantum criticality [27].

Further support for a magnetic field-induced QCP is provided by the Sommerfeld coefficient *γ*(*T*) as *T* → 0, where *γ*(*T*) = *C_el_*(*T*)/*T*. This coefficient is expected to diverge at the critical field *μ_0_H_c_*. As shown in Fig. 2b, *γ* (calculated at our lowest measured temperature of *T* = 64 mK) initially increases slowly with applied magnetic field, reaching a broad maximum of 67.3 mJ mole-Fe^−1^ K^−2^ near the critical field *μ_0_H_c_* ∼ 0.13 T, before sharply decreasing at higher fields. Although a clear divergence in *γ* was not observed due to the broad peak, the distinct *λ*-shaped curve in the *λ*–*μ_0_H* diagram conforms to the existence of a continuous second-order quantum phase transition at *μ_0_H_c_*.

During these measurements, we noticed that the value of *C_el_*(*T*)/*T* is nearly two orders of magnitude smaller than those typically found in heavy-fermion systems with LRMO, which generally reach several thousand mJ mole^−1^ K^−2^ around 0.1 K [5,6,9]. Since the low-temperature quantum critical behaviour is driven by the involvement of local moments, this relatively small *C_el_*(*T*)/*T* aligns with the unique nature of local moments in this disordered system, where Fe atoms possess varied and predominantly small (even zero at some sites) magnetic moments. Nevertheless, TiFe_0.7_Cu_0.4_Sb still qualifies as a heavy-fermion material, as it demonstrates an effective electronic mass approximately 150 times that of free electrons (see Section S6, Fig. S7) and follows the Kadowaki–Woods relationship (see Section S9, Fig. S10)—both characteristic features of heavy-fermion systems.

To further investigate the presence of a QCP, we conducted magnetocaloric effect measurements on the samples. As magnetic field *μ_0_H* was swept down, the sample temperatures were recorded simultaneously (we limited *μ_0_H* to a minimum of 0.08 T due to magnetic flux jump noise from the magnets at lower fields). Although the raw data, as shown in Fig. 2d, exhibited large noise, primarily due to the small averaged magnetic moments of the samples (around 0.05 *μ_B_* per Fe atom), a distinct minimum around *μ_0_H* = 0.10 T was observed, indicative of strong spin fluctuations and consistent with the presence of a nearby QCP. The magnetic Grüneisen parameter, *Γ_H_* = 1/*T*(*dT/dμ_0_H*), was then calculated. This parameter is expected to diverge as any QCP is approached [28], following the relationship *Γ_H_* ∝ 1/(*μ_0_H* − *μ_0_H_c_*)*^vz^*, where *v* is the correlation length exponent and *z* is the dynamical exponent. As shown in Fig. 2e, *Γ_H_* initially increases as the magnetic field decreases, reaching a broad maximum near *μ_0_H* ∼ 0.17 T, before decreasing and changing sign at lower fields. By fitting *Γ_H_* for *μ_0_H* > *μ_0_H_c_* with *μ_0_H_c_* = 0.13 T, we obtained a value of *vz* = 0.33(2) (see Section S10, Fig. S11). This value deviates from the expected *vz* = 1 or 3/2 associated with AFM or FM QPTs under the Hertz–Millis scenario involving symmetry-breaking [29], raising questions about the nature of the QCP in disordered TiFe_0.7_Cu_0.4_Sb. Moreover, we observed that the temperature dependence of *Γ_H_*(*T*) at the critical magnetic field *μ_0_H_c_* also shows divergent behaviour as inferred from the relation *Γ_H_*(*T*) = −(*dM*/*dT*)/*C_el_* (see Sections S11, Fig. S12), which provide further evidence for the existence of a magnetic-field driven QCP [28,30,31].

### Transport evidence for Kondo breakdown quantum criticality

In a dilute Kondo disordered system, Kondo screening occurs independently at each local site, and physical properties are governed primarily by the single-ion characteristic Kondo temperature, *T_K_*, which is expected to be extremely low due to disorder [32] (*T_K_* is around 0.5 K in TiFe_0.7_Cu_0.4_Sb; see Section S12, Fig. S13). As the temperature decreases below *T_K_*, Kondo screening becomes more effective and extends over larger distances, eventually leading to the development of coherent interactions among local magnetic moments. However, understanding how Kondo lattice coherence develops in such disordered systems remains a challenging open question.

In the dilute regime, resistivity *ρ*(*T*) exhibits a logarithmic increase with cooling, *ρ*(*T*) ∼ −ln*T*, characteristic of the single-ion Kondo effect. Meanwhile, the onset of coherent Kondo scattering causes metallic behaviour, with *ρ*(*T*) decreasing as the temperature lowers. Consequently, *ρ*(*T*) shows a maximum at low temperatures, marking the crossover from incoherent to coherent Kondo scattering, intuitively defined by a coherence temperature, *T_coh_*. Figure 2f shows *ρ*(*T*) for TiFe_0.7_Cu_0.4_Sb under varying magnetic fields (with *T* limited to a minimum of 0.1 K due to current-induced heating effects; see Fig. S4). Similar to the electronic specific heat *C_el_*(*T*), the resistivity *ρ*(*T*) is nearly two orders of magnitude smaller than that of other quantum critical materials. At low fields, *ρ*(*T*) initially increases and then decreases as temperature rises, resulting in a maximum in *ρ*(*T*), which indicates the onset of Kondo lattice coherence [33].

Since the emergence of quantum criticality relates closely to the Kondo coherence, we firstly focused on resistivity data below the maximum value. This restricts our analysis to a narrow temperature range, making detailed scaling analysis difficult. Here, we fitted the data to *ρ*(*T*) = *ρ*_0_ + *AT^n^*. At zero field, the power coefficient *n* is approximately 3.6, deviating from the Fermi-liquid behaviour (*n* = 2) seen in the ground state of AFM or FM materials with QCPs. As the field increases, *n* initially decreases to around 1 at *μ_0_H* = 0.2 T, and likely increases at higher fields (although *n* could not be determined for *μ_0_H* > 0.22 T, as discussed later). The minimum value of *n* = 1 in the *n*–*μ_0_H* diagram may indicate a QCP at *μ_0_H* = 0.2 T or a nearby QCP, considering the significant disorder present in the system [34].

With increasing magnetic field, the coherence temperature *T_coh_* corresponding to the maximum in ρ(T) shifts monotonically to lower temperature (indicated by the black dashed line in Fig. 2f; see Section S13, Fig. S14 for more quantitative analysis). At *μ_0_H* > 0.22 T, the maximum peak is unobservable as it shifts below 0.1 K. Similar behaviour is widely observed in other disordered Kondo system (e.g. Ce_x_La_1-x_Cu_6_ upon dilution of the magnetic sublattice [35]), indicating the destruction of coherent Kondo interactions as tuning the control parameter (magnetic field here). In the Kondo breakdown scenario, applying a magnetic field enhances Kondo screening, allowing more conduction electrons to incorporate with the local moments and form composite fermions. Consequently, local moments are increasingly screened and *T_coh_* goes to lower temperatures, consistent with the behaviour observed in TiFe_0.7_Cu_0.4_Sb.

To confirm whether the transport properties align with the Kondo breakdown scenario, we performed Hall measurements, which provide information about the Fermi surface volume. As magnetic field increases, the formation of composite fermions should result in a larger Fermi surface, as more local electrons, originally contributing to local moments, become part of it. Determining the *ρ_H_* in TiFe_0.7_Cu_0.4_Sb is challenging due to strong mixing signals from magnetoresistivity. To address this, we applied the standard anti-symmetrization process to extract ρ_H_ by sweeping the magnetic fields in both directions (note that data below *μ_0_H* = 0.1 T are truncated due to magnetic flux jump noise from the magnets; see Section S14, Fig. S15). Due to experimental restrictions, we cannot quantitatively subtract the anomalous Hall effect contribution; however, our estimation indicates that it is small compared to the dominant ordinary Hall contribution, so we continue to denote the anti-symmetrized Hall resistivity as *ρ_H_*. Figure 2g displays several representative isotherms of Hall resistivity *ρ_H_* at low temperatures. Despite the large scattering, *ρ_H_* shows a clear evolution from steep to shallow slopes as the magnetic field increases (see black dashed lines in Fig. 2g), indicating changes in the Hall coefficient and, thus, the Fermi surface.

To quantitatively analyze Hall data, we followed the method of Paschen *et al.* [36] and used $R_H^*( {{\mu }_0H} ) = R_H^\infty - ( {R_H^\infty - R_H^0} )\gamma ( {{\mu }_0H} )$ as a fitting function (see Section S15, Fig. S16). Here, $R_H^0$ is the zero-field Hall coefficient and $R_H^\infty $ is the asymptotic differential Hall coefficient at large fields. *γ*(*μ_0_H*) is a crossover function parameterized as *γ*(*μ_0_H*) = 1/[1 + (*μ_0_H*/*μ_0_H_cs_*)*^p^*], where *μ_0_H_cs_* is the crossover field and *p* is a real number. Fits of ${\rho }_H = \smallint R_H^*( H )dH$ at different temperatures, and their derivatives corresponding to $R_H^*( {{\mu }_0H} )$, are shown in the solid lines in Fig. 2g and h, respectively. At all measured temperatures, $R_H^*$ decreases monotonically from low-field to high-field. In the single-carrier model of the Hall coefficient with field-independent mobility, $R_H^*$ is inversely proportional to the Fermi surface volume (i.e. carrier density), and thus the observed decrease of $R_H^*$ with increasing magnetic fields supports the Kondo breakdown phenomenon in TiFe_0.7_Cu_0.4_Sb.

### Magnetic evidence for cluster spin-glass ground state

In most heavy-fermion metals, the onset of a magnetic field-induced QCP of the Kondo breakdown type is typically accompanied by the destruction of LRMO, i.e. the ground state at *T* → 0 and *μ_0_H* → 0 is usually either AFM or FM. However, in TiFe_0.7_Cu_0.4_Sb, due to significant disorder, LRMO is expected to be absent, making the ground state particularly intriguing.

Extensive studies have shown that the random-exchange Heisenberg-Kondo Hamiltonian effectively describes a disordered Kondo lattice (see Section S16, Fig. S17). By extending the spin-rotation symmetry to *SU*(*M*) and applying a large-*M* dynamical mean-field theory, a QPT between a spin-liquid and a heavy-fermion liquid has been identified [13,15], leading to the emergence of a Kondo breakdown QCP. However, when the spin rotation symmetry is broken and magnetic order is associated, this QPT is most likely to occur between a spin-glass and a heavy-fermion liquid, as analyzed by renormalization group [12] and quantum Monte Carlo techniques [11,16] within the context of *SU*(*2*) symmetry—a more realistic scenario potentially relevant to our findings here.

**Figure 3.**
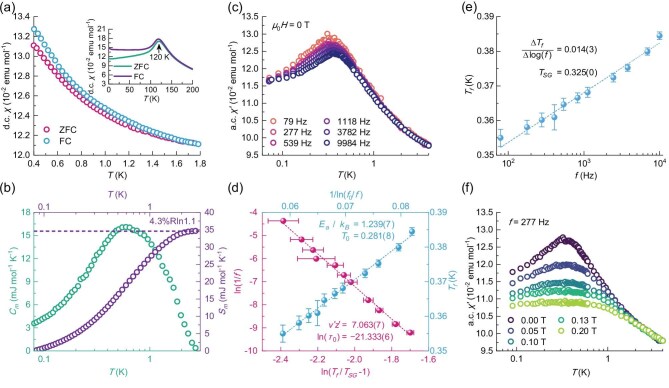
Magnetic measurements of TiFe_0.7_Cu_0.4_Sb. (a) dc magnetic susceptibility, *χ*(*T*), measured from 1.8 K down to 0.4 K. The inset shows *χ*(*T*) above 2 K, which has a peak at *T* = 120 K. (b) Zero-field magnetic specific heat coefficient, *C_m_*(*T*), and the corresponding magnetic entropy, *S_m_*. The calculated *S_m_* is approximately 4.3% of the expected value. (c) Temperature and frequency dependence of the real part of the ac susceptibility, *χ′*(*T*), measured at zero magnetic field. (d) The red data represent the linear fit of the freezing temperature as ln(*T_f_*/*T_SG_* − 1) versus ln(1/*f*). The intercept and slope provide values for ln(*τ*_0_) and *v′z′*, respectively. The blue data represent the linear fit of the freezing temperature, *T_f_*, versus frequency as 1/ln(*f*_0_/*f*). The intercept and slope yield values for *T*_0_ and *E_a_*/*k_B_*, respectively (see main text for details). (e) Frequency dependence of the freezing temperature, *T_f_*, extracted from *χ′*(*T*) by fitting peaks using a Lorentzian function. The dashed line represents the best linear fit to the datapoints, with its intercept and slope providing values for *T_SG_* and Δ*T_f_*/Δlog(*f*), respectively. (f) Magnetic field dependence of *χ′*(*T*). With increasing *μ_0_H*, the peak is gradually suppressed, indicating the weakening of the cluster spin-glass state.

To investigate whether TiFe_0.7_Cu_0.4_Sb exhibits spin-glass behaviour, we performed low-temperature zero-field-cooled (ZFC) and field-cooled (FC) dc magnetic susceptibility *χ(T)* measurements using a custom-built setup (see Section S4). As shown in Fig. 3a, the ZFC and FC curves overlap initially when cooling begins at 1.8 K. However, as the temperature decreases, the separation between the ZFC and FC branches becomes evident, providing compelling evidence for a spin-glass state. The absence of peaks in the curves indicates that the spin-freezing temperature *T_f_* is below 0.4 K.

Additionally, high-temperature ZFC and FC measurements (above 2 K, using standard techniques; see Section S4) revealed a peak at $T_f^{\prime}{\rm}$= 120 K, suggesting that a spin-glass phase also exists at high temperatures (inset of Fig. 3a). This high-temperature spin-freezing behaviour is typical in disordered systems [1], but is particularly notable in TiFe_0.7_Cu_0.4_Sb as it undergoes a two-stage freezing process of its magnetic moments: the first at $T_f^{\prime}$ = 120 K, followed by a second stage at much lower temperatures, below 0.4 K. To roughly estimate the number of active magnetic moments contributing to the low-temperature quantum critical behaviour, we calculated the low-temperature magnetic entropy, *S_m_*, from the magnetic specific heat, *C_m_* (see Section S6) through ${S}_m = \mathop \smallint \nolimits_0^T \frac{{{C}_m}}{T}dT$, as shown in Fig. 3b. The theoretical magnetic entropy for a spin-*S* particle is *S_m_* = *R*ln(2*S* + 1), assuming an average spin value of *S* ∼ 0.05, derived from saturated magnetization. However, the calculated *S_m_* is only 34.6 mJ mol^−1^ K^−1^, much lower than the theoretical value of 792.5 mJ mol^−1^ K^−1^. This indicates that nearly all magnetic moments freeze around $T_f^{\prime}$ = 120 K, with only about 4.3% actively contributing to the low-temperature quantum critical process.

To further clarify the nature of the low-temperature spin-glass phase, we performed ac susceptibility measurements, allowing us to probe much lower temperatures (down to 0.05 K). As shown in Fig. 3c, the real part of the ac susceptibility *χ′*(*T*) responds differently at various driving frequencies (the change is small due to the small magnetic moments), indicating a broad distribution of the spin relaxation time. Notably, a peak in *χ′*(*T*) is centered at the freezing temperature *T_f_* = 0.35(5) K at 79 Hz, providing a clear signature of the spin-glass phase with short-range correlations below *T_f_*. As seen in Fig. 3c, *T_f_* increases while the peak height decreases at higher driving frequencies, which are typical characteristics of spin-glasses. A common criterion for assessing the frequency dependence of *T_f_* is the relative shift in freezing temperature per decade of frequency, defined as *δT_f_* = Δ*T_f_*/[*T_f_*Δlog(*f*)], where Δ*T_f_* is the change in freezing temperature, and Δlog(*f*) is the change in the logarithm of the frequency. For TiFe_0.7_Cu_0.4_Sb, we found *δT_f_* = 0.04 for frequencies ranging from 79 to 9984 Hz (Fig. 3e), an intermediate value between those reported for canonical spin-glass systems (*δT_f_* ∼ 0.001) [37] and for non-interacting superparamagnetic systems (*δT_f_* ∼ 0.1) [38]. This *δT_f_* value characterizes the ground state as the so-called cluster spin-glass.

In a cluster spin-glass, the magnetic entities are not individual atomic spins but clusters of spins that behave collectively. In TiFe_0.7_Cu_0.4_Sb, we propose the following scenario: the randomly distributed and varied-sized atomic spins group into clusters, which is a common feature in disordered magnetic systems. As the temperature drops to $T_f^{\prime}$ = 120 K, the atomic spins inside each cluster freeze, marking the first stage of spin freezing. Each cluster, however, retains a small but non-zero net magnetic moment. At lower temperatures, these clusters themselves behave as individual magnetic entities and contribute to the quantum critical behaviour. Further cooling below *T_f_* = 0.35 K leads to the freezing of these spin clusters, resulting in the formation of a cluster spin-glass ground state as *T* → 0.

The characteristic relaxation time of a single spin flip τ_0_ can be estimated from the frequency dependence of freezing temperature *T_f_* using $\frac{1}{f} = {\tau }_0{( {\frac{{{T}_f - {T}_{SG}}}{{{T}_{SG}}}} )}^{ - z^{\prime}v^{\prime}}$, where *T_SG_* is the spin-glass temperature as frequency approaches zero, and *z′v′* is the dynamic critical exponent [1,39]. For individual spin flip processes, τ_0_ is typically around 10^−12^ to 10^−13^ s [38]. We estimated τ_0_ = 5.43 × 10^−10^ s by linear fitting $\ln ( {\frac{1}{f}} ) = \ln ( {{\tau }_0} ) - z^{\prime}v^{\prime}\ln ( {\frac{{{T}_f - {T}_{sg}}}{{{T}_{sg}}}} )$, as shown in Fig. 3d. The significantly larger τ_0_ reflects the spin-flip process in TiFe_0.7_Cu_0.4_Sb being much slower, suggesting the presence of interacting clusters rather than individual spins (note the fitted *z′v′* *=* 7.06 also aligns with other cluster spin-glass materials [40,41]). The presence of interacting clusters is further supported by the departure of frequency dependence of *T_f_* from the Arrhenius law (where *T_0_* = 0 for canonical spin-glass) and its fit to the empirical Vogel–Fulcher law, $f = {f}_0$exp$( { - \frac{{{E}_a}}{{{k}_B( {{T}_f - {T}_0} )}}} )$, where *f*_0_ is the characteristic frequency (*f*_0_ = 1/*τ*_0_), *E_a_* is the activation energy, and *T*_0_ is the Vogel–Fulcher temperature [38]. For TiFe_0.7_Cu_0.4_Sb, we obtained *T*_0_ = 0.28 K by fitting ${T}_f = \frac{{{E}_a}}{{{k}_B}}\frac{1}{{{\mathrm{ln}}( {{f}_0/f} )}} + {T}_0$ (see Fig. 3d). A non-zero value of *T*_0_ arises from spin interactions and indicates the formation of spin clusters. The proximity of *T*_0_ to *T_f_* suggests that the RKKY interaction between clusters is strong, enabling the formation of Kondo coherence at low temperatures (we also obtained a reasonable estimate of the activation energy *E_a_* ∼ 3.49 *k_B_T_f_*).

Having confirmed the cluster spin-glass nature of the ground state, we investigated the influence of applied magnetic fields on the ac susceptibility, *χ′*(*T*). Magnetic fields are expected to suppress the cluster spin-glass phase, leading to a transition to a heavy-fermion liquid phase as the system approaches and crosses the QCP at μ_0_H > μ_0_H_c_. As shown in Fig. 3f, the peak in *χ′*(*T*) gradually diminishes as the magnetic field increases, and at μ_0_H = 0.20 T, the peak flattens out, providing qualitative evidence for the suppression of the cluster spin-glass phase. This observation is consistent with the Kondo breakdown scenario (a more detailed quantitative analysis is challenging; see Section S17, Fig. S18). Additionally, our dc susceptibility measurements exhibit a similar trend, where the FC and ZFC curves completely overlap at μ_0_H = 0.13 T (see Fig. S19).

## CONCLUSION AND OUTLOOK

**Figure 4.**
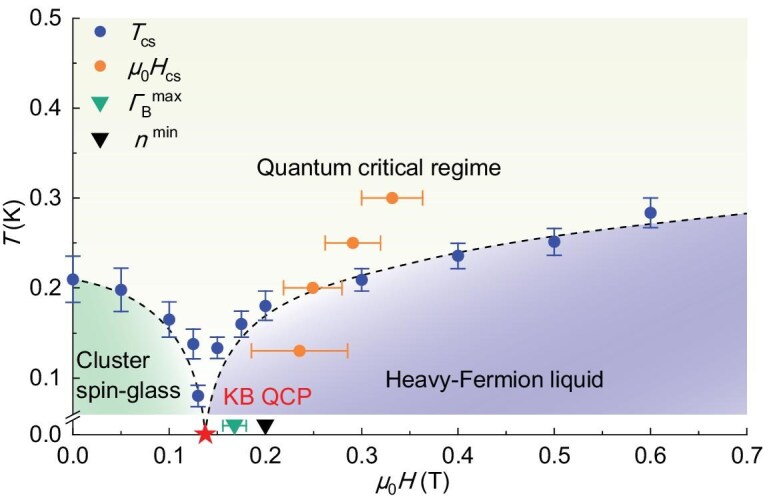
Derived phase diagram of TiFe_0.7_Cu_0.4_Sb. Blue circles represent the temperature scale *T_cs_*, derived from specific heat measurements, while orange circles indicate the magnetic field scale *μ_0_H_cs_*, obtained from Hall measurements. The green arrow points to the magnetic field position where the magnetic Grüneisen parameter reaches its maximum, while the black arrow points to the magnetic field position where the resistivity exponent *n* approaches 1. The red star indicates the proposed position of the QCP. Black dashed lines serve as visual guides. Crossing the Kondo breakdown (KB) QCP leads to a QPT from a cluster spin-glass to a heavy-fermion liquid as *T* approaches zero.

We now attempt to combine the results of our measurements into a temperature-field phase diagram, as shown in Fig. 4. A fan-shaped quantum critical regime, marked by the logarithmic temperature dependence of the electronic specific heat coefficient, is observed between the cluster spin-glass and heavy-fermion liquid phases. The phase boundaries are defined by the temperature scale *T_cs_*. Below *T_cs_*, the heavy-fermion liquid ground state forms at higher magnetic fields (μ_0_H > μ_0_H_c_), indicated by the formation of Kondo spin-singlets with large Fermi surfaces. At lower field (μ_0_H < μ_0_H_c_), RKKY fluctuations disrupt the Kondo coherent composites, while strong magnetic frustration, originating from the random distribution of magnetic atoms, prevents LRMO, resulting in a cluster spin-glass state. Although QPTs have been reported in insulating spin-glass systems [42], our work provides evidence consistent with the occurrence of a field-induced Kondo-breakdown QPT in a heavy-fermion metal with a spin-glass phase, where both itinerant electrons and local moments play essential roles.

However, several unresolved issues remain regarding TiFe_0.7_Cu_0.4_Sb. Notably, charge fluctuations have recently been recognized as crucial to Kondo breakdown QPTs [43]. Given the strong disorder in our system, these fluctuations may lead to inherently inhomogeneous states near the Kondo breakdown QPT. Furthermore, it is possible that the observed quantum critical behaviours occur in isolated ‘rare regions’ of the sample, a phenomenon known as the quantum Griffiths effect [29,44]. This effect, which often arises in disordered systems, could explain the two-stage spin-glass freezing and the small values of measured quantities. Indeed, the broad peak maximum in specific heat (Fig. 2b) and magnetic Grüneisen parameter (Fig. 2e), and discrepancies between temperature and field scales across different experiments (Fig. 4) suggest that the QPT may be a ‘smeared’ transition, indicating potential Griffiths regions in our samples. An additional source of discrepancy between temperature and field scales may arise from the polycrystalline nature of our samples [45]. To elucidate these points, more detailed experimental studies, such as the synthesis of single crystals, and theoretical characterizations of the various phases are required.

Nonetheless, our conservative interpretation suggests that a magnetic field-induced QCP can develop within a spin-glass phase without associated symmetry breaking, filling a gap in our understanding of quantum criticality (see Section S18, Fig. S20). Due to its unique lattice structure, TiFe_x_Cu_2x−1_Sb hosts randomly distributed magnetic moments (in clusters) coupled antiferromagnetically. This characteristic makes the material an intriguing platform for exploring quantum many-body physics, as posited by the Sachdev–Ye–Kitaev (SYK) model [46]. The SYK model provides an exactly solvable framework incorporating randomly distributed coupling constants, *J_ij_*. Interest in the SYK model spans high-energy and condensed matter physics, from Hawking’s quantum information paradox in black holes to the strange metals observed in heavy-fermion materials. Remarkably, TiFe_0.7_Cu_0.4_Sb exhibits a perfect linear temperature dependence in resistivity at temperatures well above room temperature of 300 K (Fig. S5). This ‘bad metal’ behaviour is also one of the predictions of the SYK model.

## Supplementary Material

nwaf483_Supplemental_File

## Data Availability

The data that support the findings of this study are available from the Corresponding author upon reasonable request.
