# Supplementary material for: Emerging quantum critical phase in a cluster spin-glass
Source: Natl Sci Rev. 2025 Nov 6;13(3):nwaf483. doi: 10.1093/nsr/nwaf483 (PMC12964121; doi:10.1093/nsr/nwaf483)
Supplement: nwaf483_Supplemental_File [file nwaf483_supplemental_file.docx]

Supplementary Information for

**Emerging quantum critical phase in a cluster spin-glass**

Fang Zhang^#^, Tao Feng^#^, Yurong Ruan, Xiaoyuan Ye, Bing Wen, Liang Zhou,

Minglin He, Zhaotong Zhuang, Liusuo Wu, Hongtao He, Peijie Sun,

Zhiyang Yu^*^, Weishu Liu^*^ & Wenqing Zhang^*^

*^#^These authors contributed equally to this work.*

**Corresponding authors:* [*yuzyemlab@fzu.edu.cn*](mailto:yuzyemlab@fzu.edu.cn)*,* [*liuws@sustech.edu.cn*](mailto:liuws@sustech.edu.cn)*;* [*zhangwq@sustech.edu.cn*](mailto:zhangwq@sustech.edu.cn)

**Sec. 1: Materials preparation**

**Sec. 2: Structure characterization**

**Sec. 3: Ab initio calculations**

**Sec. 4: Thermodynamical, transport, and magnetic measurements**

**Sec. 5: Substruction of the nuclear Schottky contribution to the specific heat**

**Sec. 6: Substruction of the phonon contribution to the specific heat**

**Sec. 7: Substruction of the magnetic Schottky anomaly to the specific heat**

**Sec. 8: Determination of scale** $\boldsymbol{T}_{\boldsymbol{cs}}$ **in electronic specific heat**

**Sec. 9: Kadowaki-Woods relation**

**Sec. 10: Fitting of the magnetic Grüneisen parameter**

**Sec. 11: Temperature dependence of the magnetic Grüneisen parameter**

**Sec. 12: Estimation of the Kondo temperature**

**Sec. 13: Crossover between coherent and dilute regimes**

**Sec. 14: Extracting the Hall resistivity** $\boldsymbol{\rho}_{\boldsymbol{H}}$ **from the raw data**

**Sec. 15: Quantitative analysis of the crossover functions in Hall measurements**

**Sec. 16: Effective Hamiltonian of TiFe_x_Cu_2x-1_Sb**

**Sec. 17: ac and dc magnetic susceptibility at finite magnetic fields**

**Sec. 18: Schematic of global phase diagram**

**Sec. 1: Materials Preparation**

The polycrystalline TiFe_0.7_Cu_0.4_Sb samples were synthesized through a combined approach of arc melting, mechanical alloying, and spark plasma sintering (SPS). The raw materials—Ti (99.99% pure rods), Fe (99.98% pure sheets), Cu (99.9% pure rods), and Sb (99.99% pure rods)—were weighed according to the nominal composition of TiFe_0.7_Cu_0.4_Sb. Firstly, the alloy ingot was prepared by arc melting on a water-cooled copper hearth under an Ar-protected atmosphere. The ingot was flipped over and re-melt for four times to ensure better homogeneity. A small amount of extra Sb was added to compensate the weight loss of Sb due to its high vapor pressure. Then the ingot was loaded into a stainless-steel ball milling jar in a glove box under an Ar atmosphere with an oxygen level of $<$1 ppm. After ball milling for 12 min in a SPEX 8000M mixer, the ball-milled powders were loaded into a graphite die with an inner diameter of 12.7 mm, in the glove box. The graphite die with the loading powder was immediately sintered at 750°C under a pressure of 50 MPa for 5 min via spark plasma sintering (SPS) (SPS-211Lx, Fuji Electronic Industrial Co. LTD).

**
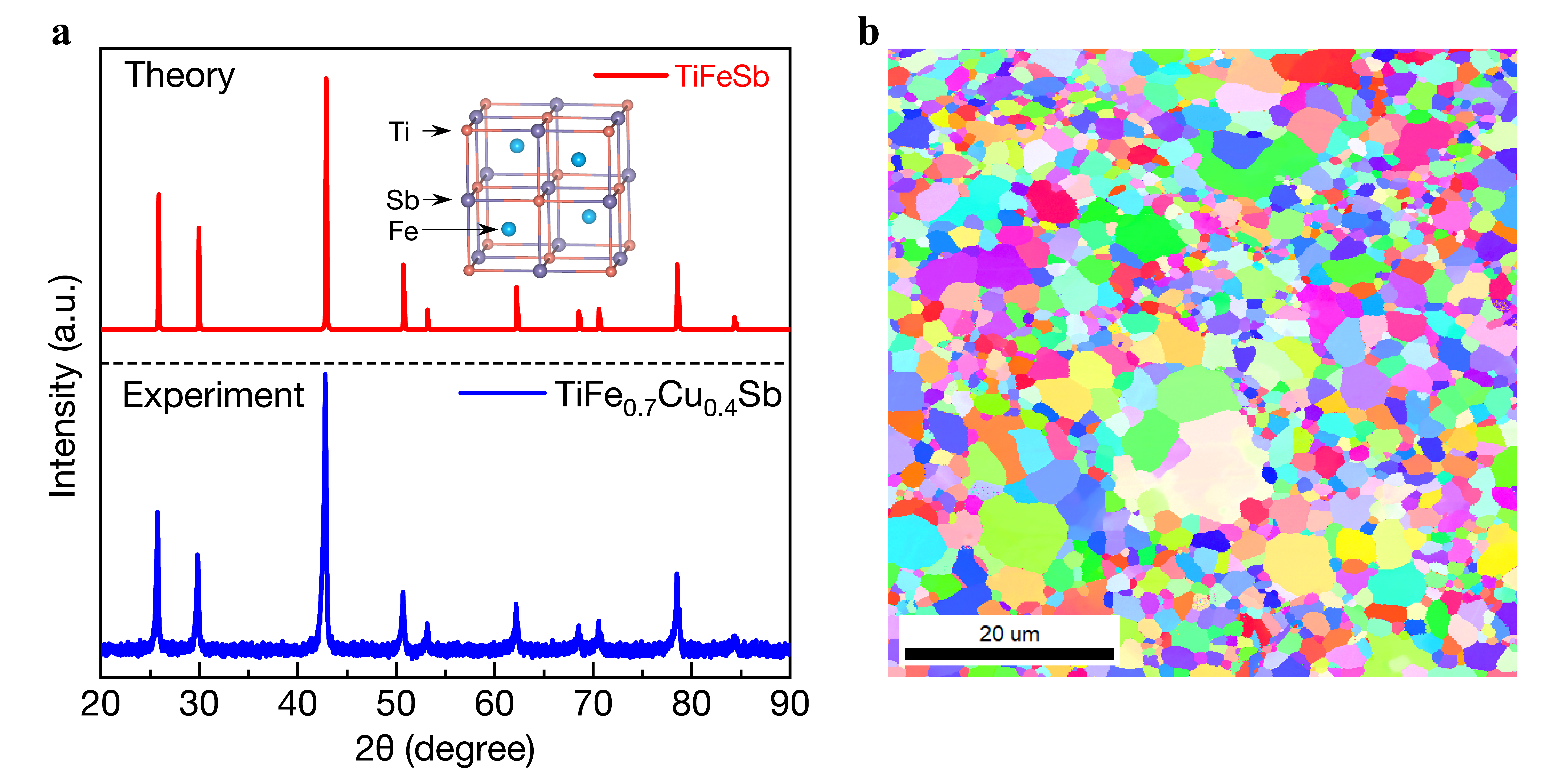
**

**Fig. S1 | XRD and EBSD patterns of TiFe_0.7_Cu_0.4_Sb. a,** The upper panel shows the theoretical X-ray diffraction (XRD) pattern of TiFeSb, a half-Heusler material where Fe occupies all 4c sites. The inset depicts the crystal structure. The lower panel displays the experimental XRD pattern of TiFe_0.7_Cu_0.4_Sb, confirming phase purity and the absence of impurities. **b**, The electron backscatter diffraction (EBSD) pattern of polycrystalline TiFe_0.7_Cu_0.4_Sb, highlighting the size and distribution of the grains.

**Sec. 2: Structure characterization**

The phase purity of the product was measured by powder X-ray diffraction (XRD) on a Rigaku D/Max-2550 instrument (Cu K$\alpha$ radiation, $\lambda=$ 1.5418 Å, 18 KW). The microstructures of the samples were examined by a high-resolution transmission electron microscopy (HRTEM) (JEM-F200, JEOL, Japan) and a probe Cs-corrected TEM (Themis ETEM, Thermo Fisher Scientific, USA). TEM specimens were prepared by mechanical slicing, polishing, and dimpling, followed by ion-milling. Energy-dispersive spectroscopy (EDS) was used to determine the distribution of elements at the nanoscale.





**Fig. S2 | Microstructures of TiFe_0.7_Cu_0.4_Sb. a**, High-angle annular dark-field scanning transmission electron microscopy (HAADF-STEM) image of TiFe_0.7_Cu_0.4_Sb. **b**, corresponding energy dispersive spectroscopy (EDS) maps showing the uniform distribution of Ti, Fe, Cu, and Sb elements within the sample. **c**, HAADF-STEM image along the [110] direction. **d**, integrated differential phase contrast (iDPC) image along the [110] direction.

**Sec. 3: Ab initio calculations**

Density functional theory (DFT) calculations were performed using the Vienna Ab initio Simulation Package (VASP)^47,48^. TiFe_0.7_Cu_0.4_Sb compound was calculated through the employment of a 3 × 3 × 3 supercell Ti_108_Fe_76_Cu_44_Sb_108_, where Fe and Cu atoms are randomly distributed along the 4c and 4d sites. The exchange-correlation functional adopted the Strongly Constrained and Appropriately Normed (SCAN)^49^. For the Brillouin zone integration, a gamma-centered 2 × 2 × 2 **k**-point mesh was selected, with a cutoff energy set at 400 eV. All atoms were fully relaxed until the residual forces acting on each atom diminished below 0.02 eV/Å. An energy convergence criterion of 10^-6^ eV was set to guarantee the attainment of highly converged results.

**
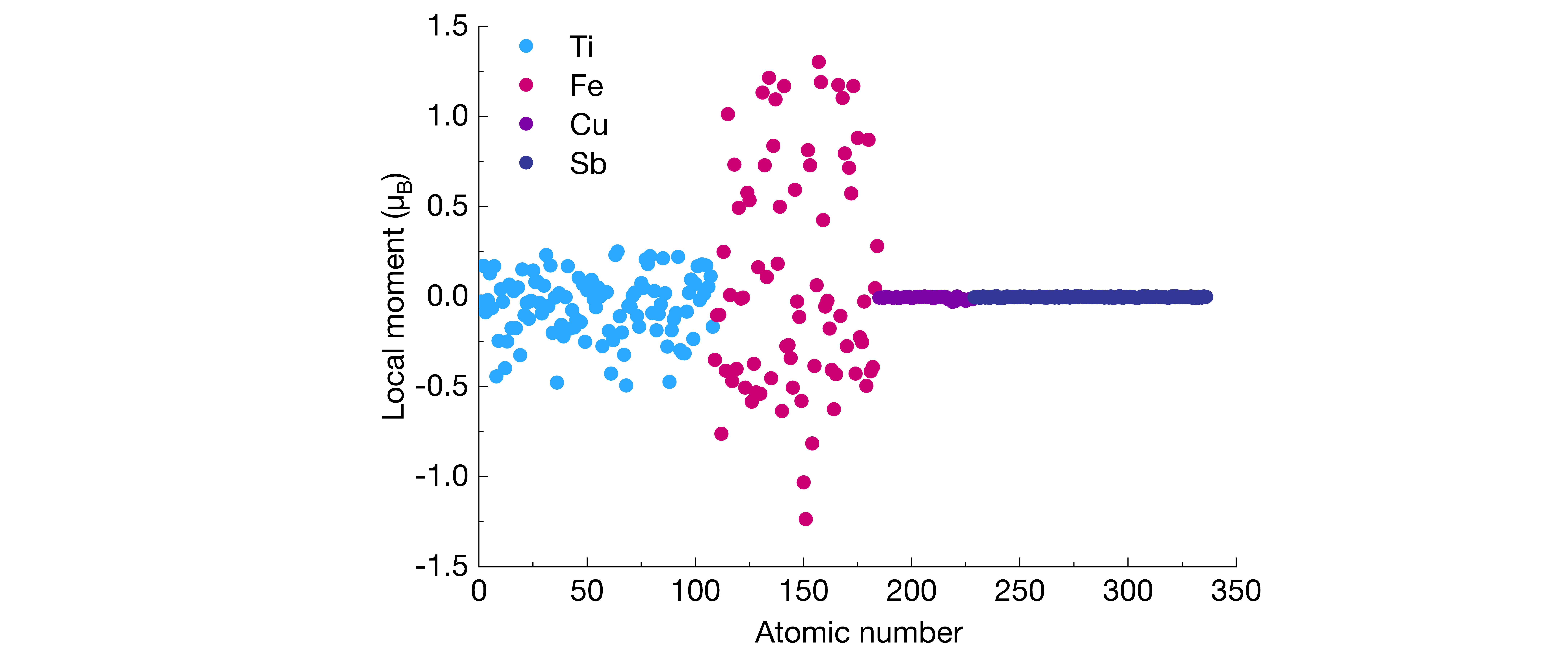
**

**Fig. S3 | Calculated distribution of magnetic moments in TiFe_0.7_Cu_0.4_Sb.** Ab initio calculations revealed that magnetic moments primarily originate from Fe atoms, with a minor contribution from Ti atoms. Due to strong disorder, Fe atoms experience varying crystal field environments, leading to a range of magnetic moments between 0 and 1.5 $\mu_{B}$.

**Sec. 4: Thermodynamical, transport, and magnetic measurements**

The heat capacities were performed by Quantum Design Physical Property Measurement System (PPMS, 14T) with a dilution refrigerator (DR) insert in the temperature range of 0.05 K to 4 K at a set of fixed magnetic fields from 0 to 0.6 Tesla. The sample was mounted to the sample platform with a small amount of Apiezon grease and measured with the standard relaxation method.

The magnetocaloric effect (MCE) was evaluated by using a homemade quasi-adiabatic sample stage adapted for the Oxford ^3^He refrigerator. The sample stage consists of a 3×3 mm² sapphire plate suspended by thin nylon wires within a PEEK frame. A resistance thermometer (CX-1010 bare chip sensor, Lake Shore Cryotronics Inc.), fixed to the sapphire plate and connected by 25 $\mu$m diameter manganin wires, was used to monitor the temperature changes during MCE measurement.

A lock-in technique was applied to measure the resistivity, magnetoresistivity and Hall resistivity. The sample was loaded on the platform of DR insert of PPMS, with the temperature range between 0.05 K and 4 K. For resistivity and magnetoresistivity measurements, the sample was bonded using the four-contact configuration, and a relatively large current of 150 $\mu$A is used due to the small value of the resistivity. Because of the heating effects of the current, the minimum temperature we can reach is near 0.1 K instead of the designed 0.05 K. For Hall effect measurement, the sample was bonded as standard Hall bar. The ac current was set with an amplitude of 100 $\mu$A and a frequency of 37 Hz. Due to the magnetic flux jump noise of magnets, the minimum magnetic fields $\mu_{0}H$ we present is 0.1 T, as the temperature is not stable for smaller fields.

The low-temperature (0.4 K to 1.8 K) dc magnetic susceptibility was measured by using a vibrating sample magnetometer equipped with a SQUID sensor (SQUID-VSM, Quantum Design) in conjunction with a ^3^He insert (iHelium3). The high-temperature (above 2 K) dc magnetic susceptibility was measured by the VSM option of PPMS with an applied magnetic field of 0.01T. The ac magnetic susceptibility was performed on ACDR option of PPMS in the temperature range between 0.05 K and 4 K at a set of fixed frequency from 79 Hz to 9984 Hz. The AC Drive was set as 3 Oe.


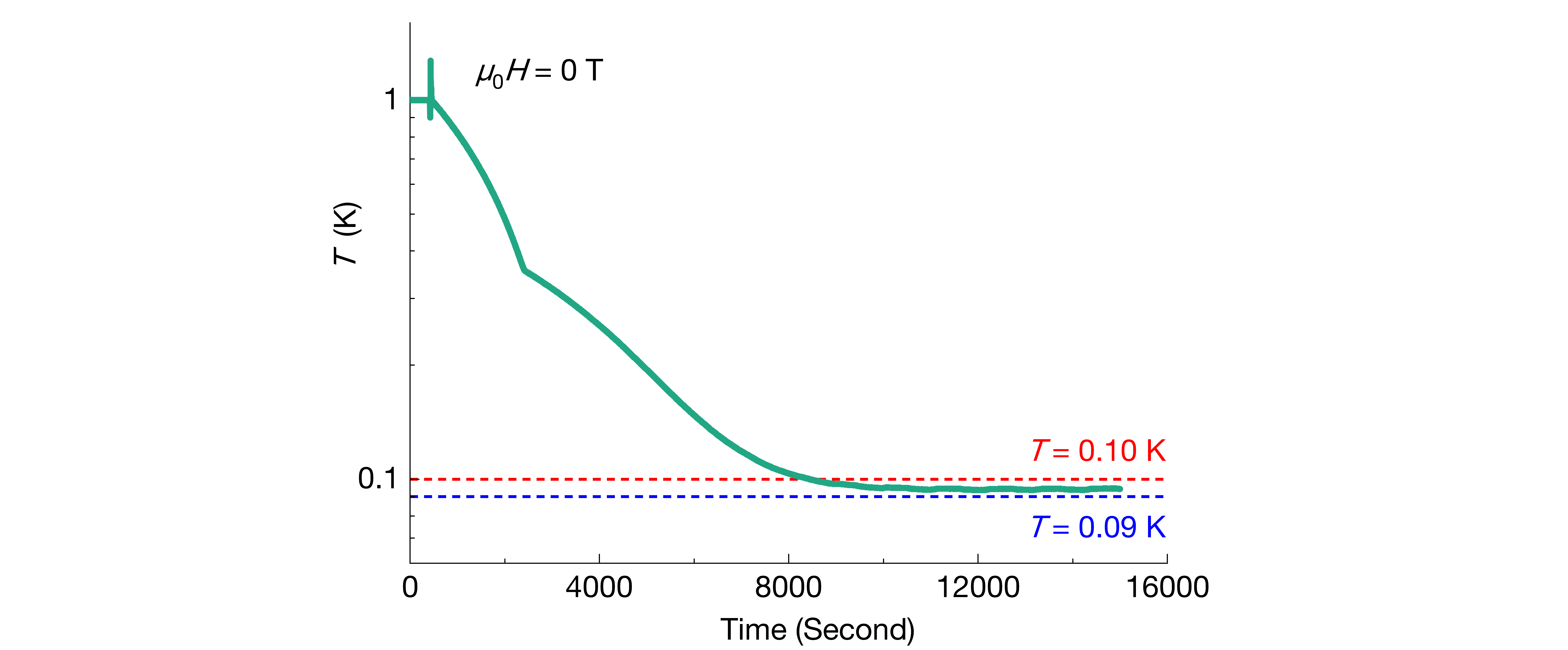


**Fig. S4 | Temperature record.** A typical temperature record during resistivity measurement. Due to heating from the applied current, the minimum achievable temperature is around 0.10 K.

**
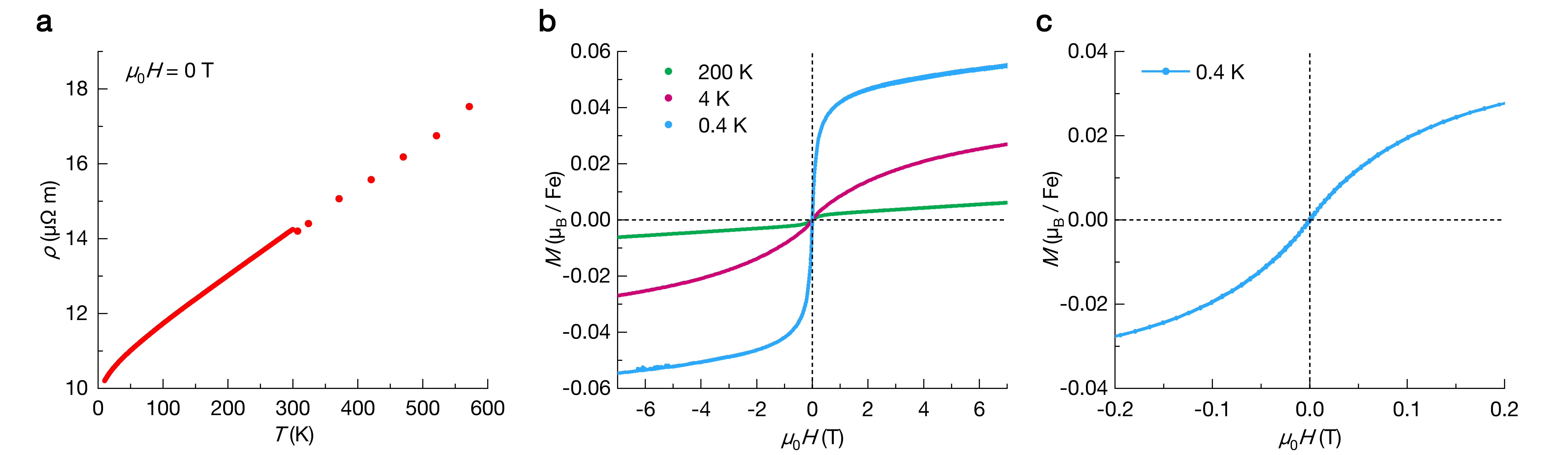
**

**Fig. S5 | High-temperature resistivity and field-dependent magnetization. a**, The resistivity from 2 K to 600 K at zero field shows metallic behaviour. A linear temperature dependence is observed from 100 K to 600 K, characterizing TiFe_0.7_Cu_0.4_Sb as a “bad metal”. **b**, Magnetization at 0.4 K, 4 K, and 200 K. With increasing the magnetic fields, magnetization increases and saturates at approximately 0.05 $\mu_{B}$ per Fe atom at 0.4 K and 5.0 T. **c**, Magnetization loops at 0.4 K, which shows no hysteresis, ruling out the ferromagnetic ordering in the system.

**Sec. 5: Substruction of the nuclear Schottky contribution to the specific heat**

As shown in Fig. S6, the raw specific heat data $C_{tot}\left( T \right)$ without any correction for zero and finite fields exhibits a sharp increase at very low temperatures on cooling. This phenomenon may be caused by the spin-glass melting down, or a nuclear Schottky contribution. However, the real part of ac susceptibility $\chi^{'}(T)$ smoothly decreases on cooling (Fig. 3c in main text), ruling out the spin-glass melting down for which would result in an increasing of the spin susceptibility. The nuclear Schottky anomaly is due to the nuclear level of Fe atoms. Even at zero magnetic field, the existence of strong lattice disorder distorts the tetrahedron crystal field of Fe, creating finite electrical field gradient that splits its nuclear levels.

Generally, the nuclear Schottky contribution to the specific heat $C_{nuc}\left( T \right)$ is

$$\begin{aligned} C_{nuc}(T)=Nk_{B}\frac{\alpha^{2}}{4I^{2}}\left[ \frac{1}{\sinh^{2}\left( \frac{\alpha}{2I} \right)}-\frac{\left( 2I+1 \right)^{2}}{\sinh^{2}\left( \frac{\left( 2I+1 \right)\alpha}{2I} \right)} \right], \alpha=\frac{A_{hf}\mu I}{\mu_{B}g_{I}T}\#\left( S1 \right) \end{aligned}$$

where $N$, $k_{B}$ and $\mu_{B}$ are Avogadro’s number, Boltzmann’s constant, and Bohr’s magneton. $A_{hf}$, $\mu$, $I$ and $g_{I}$ are hyperfine coupling, Fe-ion magnetic moment, nuclear spin, and Lande’s $g$-factor. Since these parameters are mostly unknown, direct estimation of the $C_{nuc}\left( T \right)$ based on eq. S1 is unavailable.

We estimate $C_{nuc}\left( T \right)$ following the method in ref^50^. Firstly, to a good approximation, eq. S1 can be expressed as^51^

$$\begin{aligned} C_{nuc}(T)\approx\frac{A}{T^{2}}\#\left( S2 \right) \end{aligned}$$

Then, we estimated the coefficient $A$ by linear-fitting the low-temperature $C_{tot}\left( T \right)$ with $C_{tot}\left( T \right)/T\sim A/T^{3}$ at $T\leq$ 0.1 K regions. The fitted $A$ is summarized in table S1 with standard error. All fits have a confidence interval greater than 99.7%.

The Sommerfeld coefficient $\gamma\left( T \right)$ at $T\to$ 0 reported in Fig. 2b is obtained by $\gamma\left( T \right)=\left[ C_{tot}\left( T \right)-C_{nuc}\left( T \right) \right]/T$ calculated at $T=$ 64 mK, whereas the large error bar comes from the fittings of coefficient $A$. At this low temperature, the phonon and magnetic Schottky contribution to the specific heat is negligible (as will be described next).

**
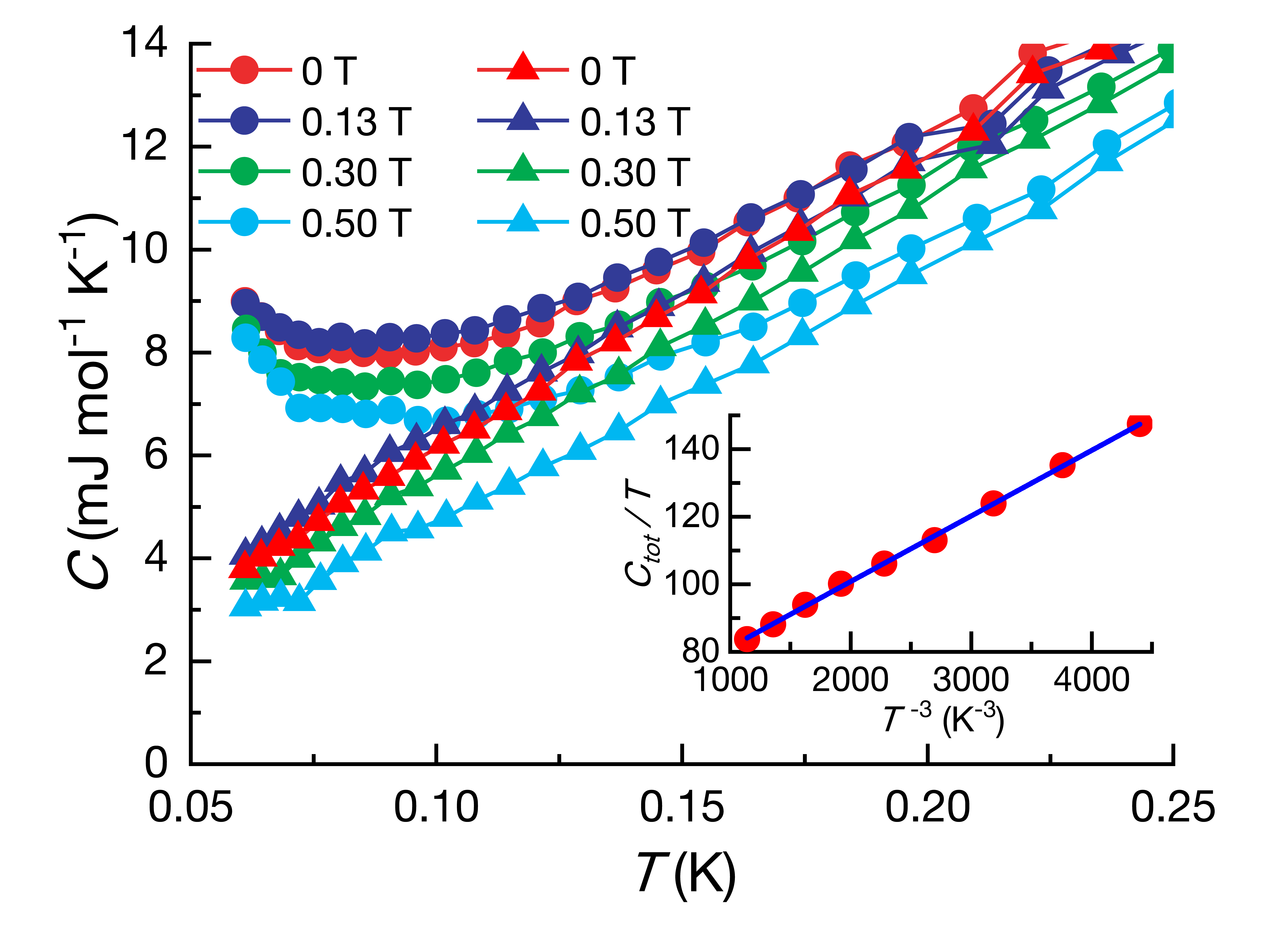
**

**Fig. S6 | Substruction of the nuclear contribution** $\boldsymbol{C}_{\boldsymbol{nuc}}$ **to specific heat.** The raw specific heat data (circles), $C_{tot}$, shows a sharp increase at low temperatures on cooling, potentially due to a nuclear Schottky contribution. After subtracting this contribution, the corrected specific heat $C_{tot}-C_{nuc}$ is plotted (triangulars). The inset shows $C_{tot}/T$ versus 1$/T^{3}$ in the $T\leq$ 0.1 K region, with a linear fit to the data.

**Sec. 6: Substruction of the phonon contribution to the specific heat**

In normal metals, the low-temperature specific heat is contributed by conduction electron and phonon as $C\left( T \right)=\alpha T+\beta T^{3}$^52^. If plotted as $C\left( T \right)/T \sim T^{2}$, the result should be a line. However, as shown in Fig. S7, the specific heat $\left[ C_{tot}\left( T \right)-C_{nuc}\left( T \right) \right]/T$ in TiFe_0.7_Cu_0.4_Sb, where the nuclear Schottky contribution has been subtracted, is a line with respect to $T^{2}$ at high-temperature regions but deviates extensively at low temperature, which is a typical characteristic of heavy-fermion materials. The low-temperature anomaly is attributed to magnetism, namely the interaction between conduction electrons and local moments, as $C_{m}\left( T \right)$.

The phonon contribution to the specific heat $C_{pho}\left( T \right)$ is obtained by linear-fitting $\left[ C_{tot}\left( T \right)-C_{nuc}\left( T \right) \right]/T$ with respect to $T^{2}$ at $T>$ 2.5 K at $\mu_{0}H=$ 0 T, and the fitted value is $\beta=$ 0.00105$\pm$0.00006 J mole-Fe^-1^ K^-4^. Since magnetic fields do not affect the crystal structure and hence $C_{pho}$, the same $\beta$ was employed at finite fields.

The intercept obtained in above fitting procedure is the contribution of normal conduction electrons, i.e., $\alpha T$ with $\alpha=$ 0.01850$\pm$0.00067 J mole-Fe^-1^ K^-2^.

The magnetic contribution to specific heat $C_{m}$ as plotted in Fig. 3b is obtained by $C_{m}\left( T \right)=C_{tot}\left( T \right)-C_{nuc}\left( T \right)-C_{pho}\left( T \right)-\alpha T$.

In a free electron model, $C_{el}=\frac{3}{2}R\left[ \frac{1}{3}\pi^{2}\frac{T}{T_{F}} \right]=\gamma_{0}T$, and $\frac{m^{*}}{m}=\frac{\gamma}{\gamma_{0}}$. In TiFe_0.7_Cu_0.4_Sb, $\gamma\approx$ 62 mJ mole-Fe^-1^ K^-2^ at $T\approx$ 0.08 K. If we approximate the Fermi temperature to ${10}^{5}$ K, which is a typical value for metals, the low temperature effective mass of TiFe_0.7_Cu_0.4_Sb is estimated to be 151 times to that of free electrons, suggesting TiFe_0.7_Cu_0.4_Sb is a heavy-fermion metal.


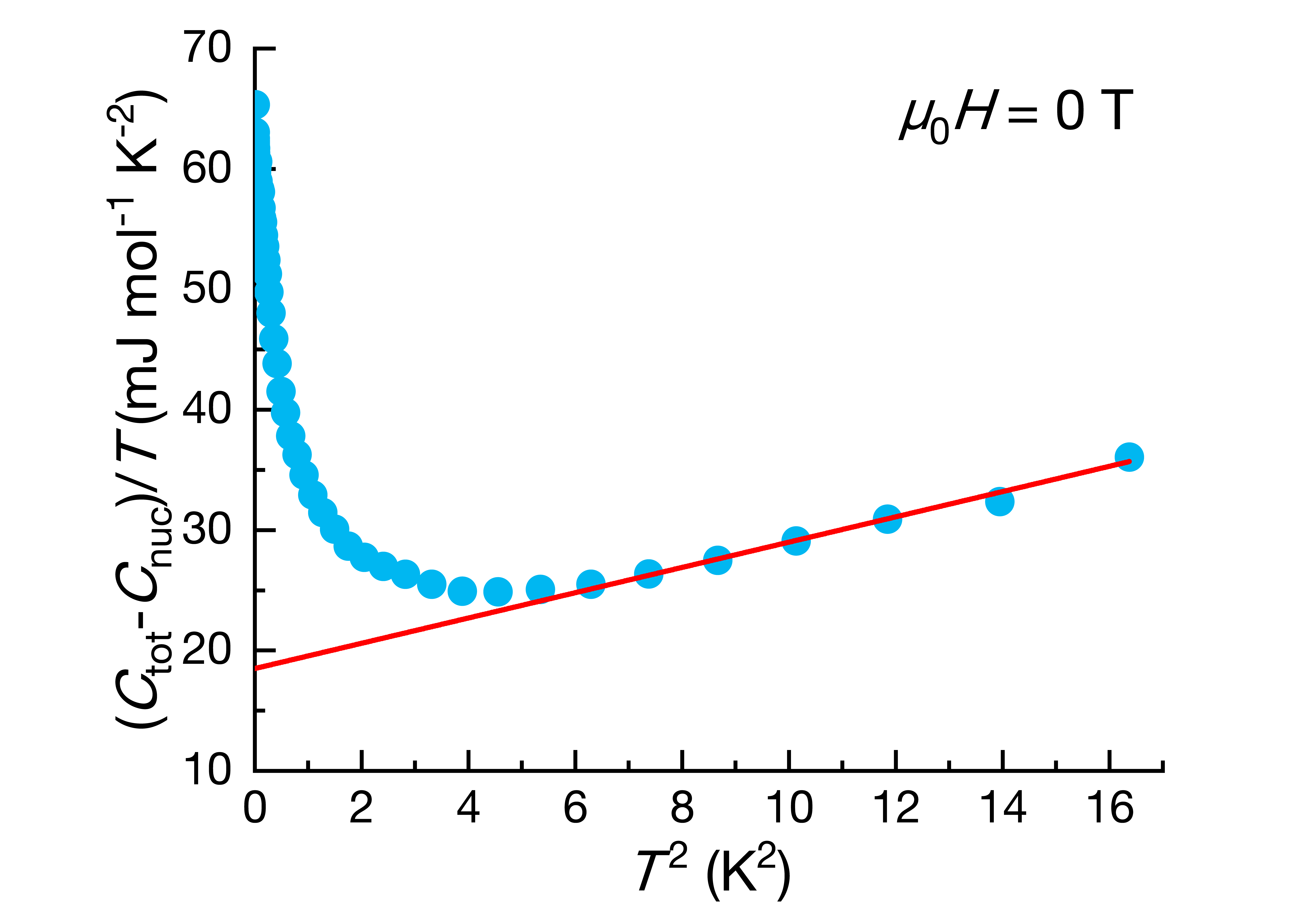


**Fig. S7 | Substruction of the phonon contribution** $\boldsymbol{C}_{\boldsymbol{pho}}$ **to specific heat.** The circles represent the specific heat after the subtraction of the nuclear contribution, $C_{tot}-C_{nuc}$. At higher temperatures, the relation $C_{tot}-C_{nuc}=\alpha T+\beta T^{3}$ holds, with the red line indicating the best fit above 2.5 K ($\alpha=$ 0.01850$\pm$0.00067 J mole-Fe^-1^ K^-2^, $\beta=$ 0.00105$\pm$0.00006 J mole-Fe^-1^ K^-4^). The low-temperature deviation signifies the presence of magnetic specific heat, $C_{m}$, due to spin fluctuations. $C_{m}$ can be obtained by $C_{m}=C_{tot}-C_{nuc}-\alpha T-\beta T^{3}$, where $C_{pho}=\beta T^{3}$.

**Sec. 7: Substruction of the magnetic Schottky anomaly to the specific heat**

In Fig. 2a, we plotted the electronic specific heat coefficient $C_{el}(T)$. In addition to the nuclear Schottky contribution $C_{nuc}\left( T \right)$ and the phonon contribution $C_{pho}\left( T \right)$ to the total specific heat $C_{tot}\left( T \right)$, there is also a magnetic Schottky anomaly $C_{sch}\left( T \right)$ arising from the Kondo effect, which is related to single-ion impurity scattering. Specifically, $C_{sch}\left( T \right)$ can be calculated as follows:

$$\begin{aligned} C_{sch}(T)=n\frac{2N\Delta^{2}}{k_{B}T^{2}}\frac{e^{\Delta/{k_{B}T}}}{\left( 1+2e^{\Delta/{k_{B}T}} \right)^{2}}\#\left( S3 \right) \end{aligned}$$

where $N$ and $k_{B}$ are Avogadro’s number and Boltzmann’s constant, respectively. Here, $\Delta$ is the energy level splitting, and $n$ counts how many local moments (per mole-Fe) contributes to $C_{sch}$.

In TiFe_0.7_Cu_0.4_Sb, due to the presence of strong disorder, the local moments (mostly originating from Fe atoms) vary from 0 to 1.5 $\mu_{B}$. In the main text, we take an average value of 0.05 $\mu_{B}$ as obtained from magnetization measurements. To estimate the parameters $n$ and $\Delta$ (based on a two-energy level system assumption), we take a more simplified approach, assuming that the local moments can only take values of either 0 or 1.5 $\mu_{B}$. Only Fe atoms with a magnetic moment of 1.5 $\mu_{B}$ contribute to $C_{sch}$, while those with zero magnetic moment do not. In this physical model, each Fe atom either contributes a finite and same value to $C_{sch}(T)$ or none at all, which simplifies the estimation.

We first estimate $\Delta$. According to this model, the broad peak $C_{m}(T)$ at $T^{+}=$ 0.60 K (see Fig. 3b in the main text) is attributed to the Kondo effect of these magnetic Fe atoms with a moment of 1.5 $\mu_{B}$. Therefore, we estimate the energy splitting $\Delta$ by finding $\left. \frac{\partial C_{sch}}{\partial T} \right|_{T=T^{+}}=0$, leading to:

$$\begin{aligned} 2e^{\Delta/{k_{B}T^{+}}}=\frac{\Delta/{k_{B}T^{+}}+2}{\Delta/{k_{B}T^{+}}-2}\#\left( S4 \right) \end{aligned}$$

For a rough estimate of Eq. S4, $\Delta\geq2k_{B}T^{+}$. The actual value of $\Delta$ is calculated numerically and summarized in Table S1. Increasing magnetic fields gradually enhance the Kondo coupling strength $J_{K}$ due to the increasing of spin fluctuation, resulting in a monotonic increase in $\Delta$.

The value of $n$ is roughly estimated by calculating the magnetic entropy. Specifically, for a spin- $S$ particle, the theoretical magnetic entropy is $S_{m}^{0}$ $=R\ln\left( 2S+1 \right)$ if all local moments are considered. At $\mu_{0}H=0$ T, the calculated magnetic entropy $S_{m}$ is 34.6 mJ mol^-1^ K^-1^, as elaborated in the main text. Here, for $S=$ 3/2, corresponding to a magnetic moment of 1.5 $\mu_{B}$, the theoretical magnetic entropy is 11520 mJ mol^-1^ K^-1^. Thus, for a rough estimate,

$$\begin{aligned} n \sim\frac{S_{m}}{S_{m}^{0}}=0.3\%\#\left( S5 \right) \end{aligned}$$

This indicates that, if all magnetic Fe ions had moments of 1.5 $\mu_{B}$, only approximately 0.3% of the Fe atoms contribute to the magnetic moments at low temperatures, while the remainder are nonmagnetic. The reason for this small fraction of magnetic Fe is due to the following effects: (1) Strong disorder in the system results in different crystal field environments for each Fe ion. Some Fe ions experience a crystal field environment similar to that found in half-Heusler (non-magnetic) or full-Heusler (weakly magnetic) compounds, resulting in very small magnetic moments; (2) As discussed in the main text, spin-glass freezing occurs at $T_{f}^{'}=$ 120 K. Consequently, at low temperatures (below 1 K), most spins are frozen and do not participate in the quantum critical process.

The actual value of $n$ used under different magnetic fields are summarized in table S1. Considering the uncertainty in obtaining $C_{m}$ by subtracting other contributions to specific heat and the potential underestimation of $S_{m}$ due to a finite temperature range, the values of $n$ used are very close to 0.3% (ranges from 0.28% to 0.45% with increasing fields) but not exactly equals to it. The actual values of $n$ were determined by subtracting the peak at ${T=T}^{+}$ in $C_{el}\left( T \right)$, where $C_{el}\left( T \right)=C_{tot}\left( T \right)-C_{nuc}\left( T \right)-C_{pho}\left( T \right)-C_{sch}\left( T \right)$. $C_{el}\left( T \right)$ at different magnetic fields is plotted in Fig. S8.


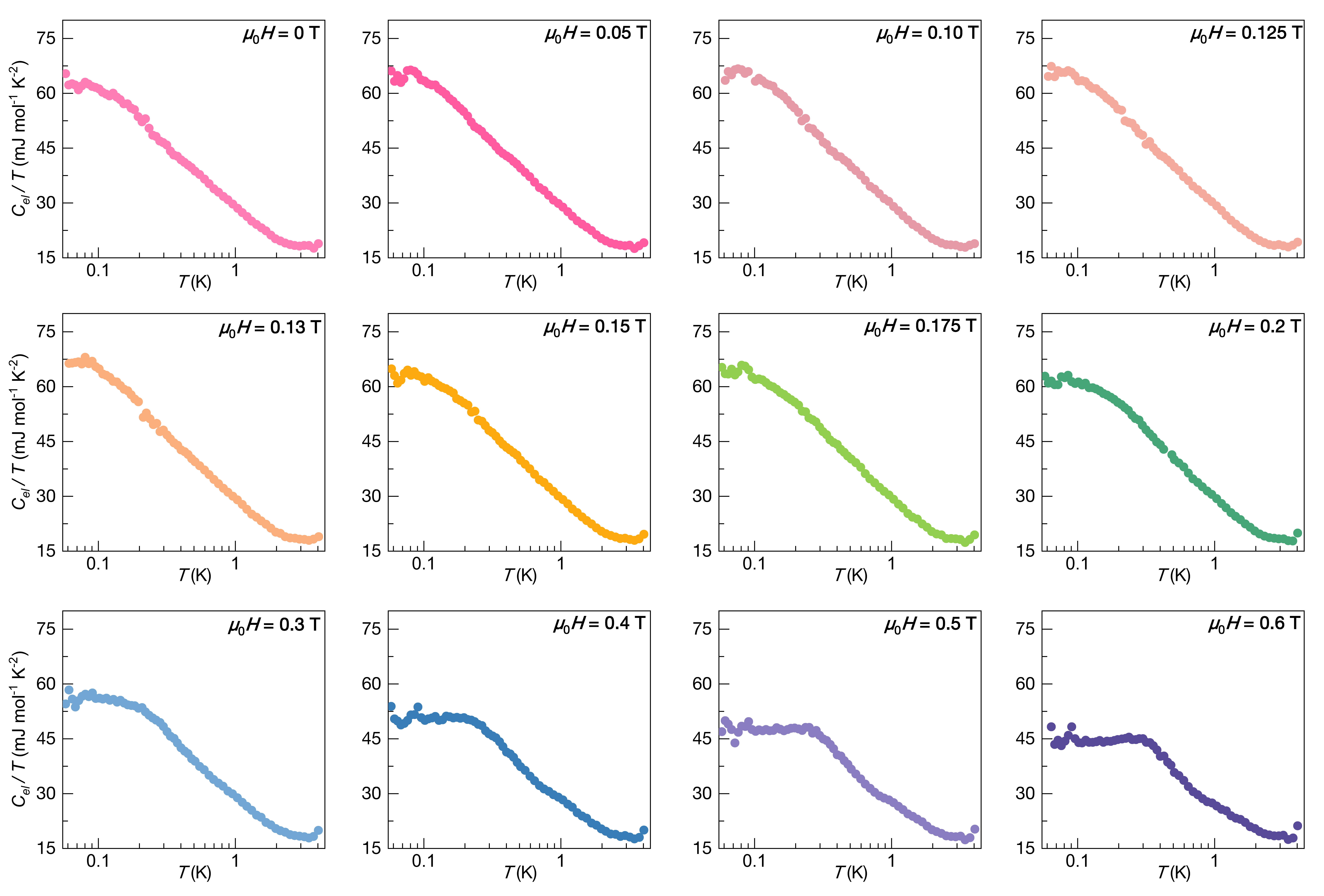


**Fig. S8 | Electronic specific heat coefficient** $\boldsymbol{C}_{\boldsymbol{el}}$**.** The electronic specific heat coefficient is determined as $C_{el}=C_{tot}-C_{nuc}-C_{pho}-C_{sch}$, plotted as $C_{el}/T$ for different magnetic fields.

**Sec. 8: Determination of scale** $\boldsymbol{T}_{\boldsymbol{cs}}$ **in electronic specific heat**


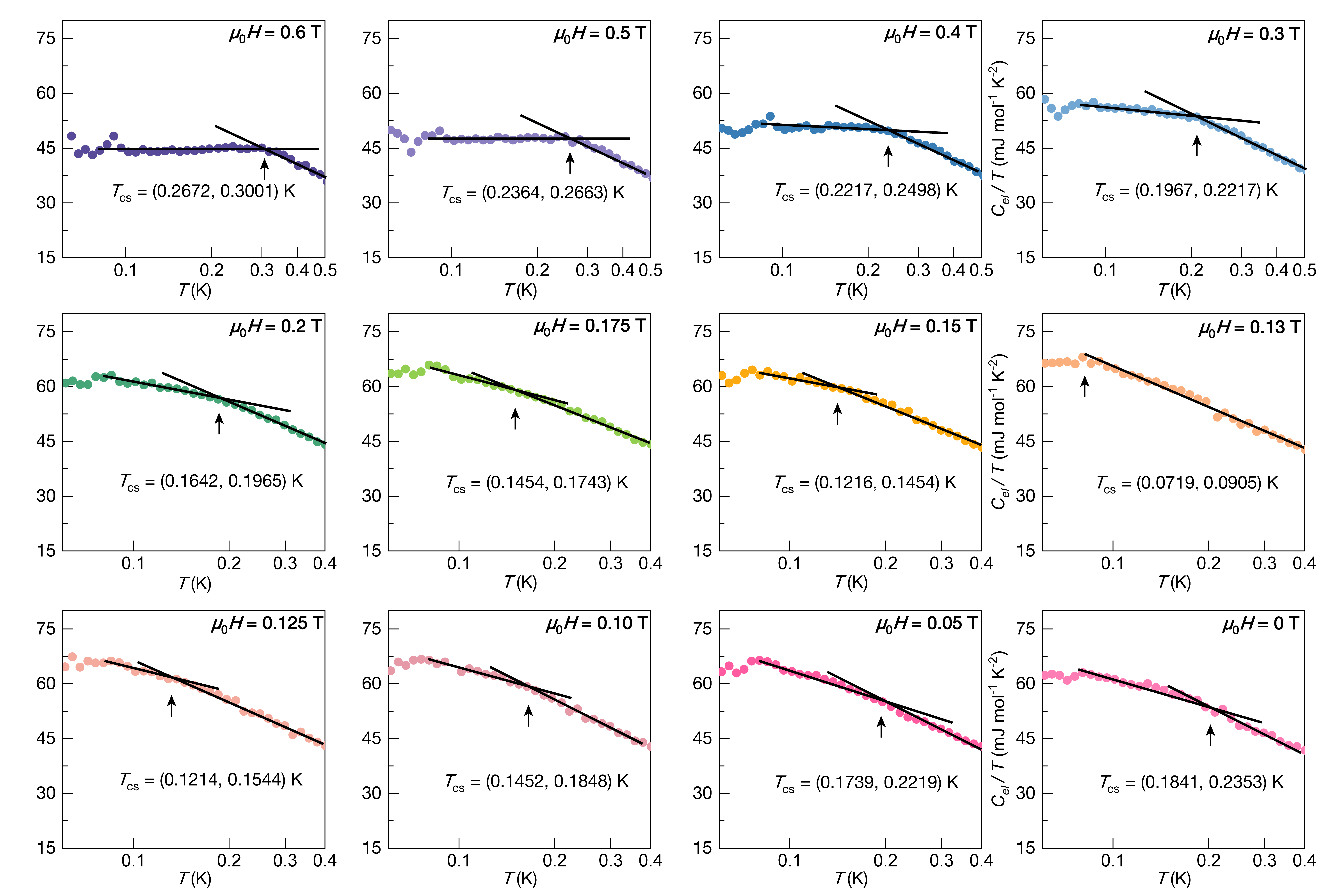


**Fig. S9 | Determination of scale** $\boldsymbol{T}_{\boldsymbol{cs}}$**.** Above and below $T_{cs}$, $C_{el}/T$ can be fitted by the logarithmic form $C_{el}/T=C_{el}^{0}+A\log(T)$, but with different values of $A$. Below $T_{cs}$, the absolute value of $|A|$ is smaller. To determine $T_{cs}$, we try several trial temperatures $T_{cs}^{0}$ and perform fits to search for the temperature that yields high adjusted R-squared values in both the high- and low-temperature regions. This temperature is then determined to be $T_{cs}$. Due to data scattering below 0.08 K (partly due to uncertainties in the nuclear Schottky contribution, $C_{nuc}$), we only consider the temperature regime $T\geq$ 0.08 K during the fitting process. Additionally, because of the limited number of data points, there may be cases where only one trial temperature provides a satisfactory fit. In such situations, we define the uncertainty in $T_{cs}$ by the temperature step size in our measurements.

**Sec. 9: Kadowaki-Woods relation**


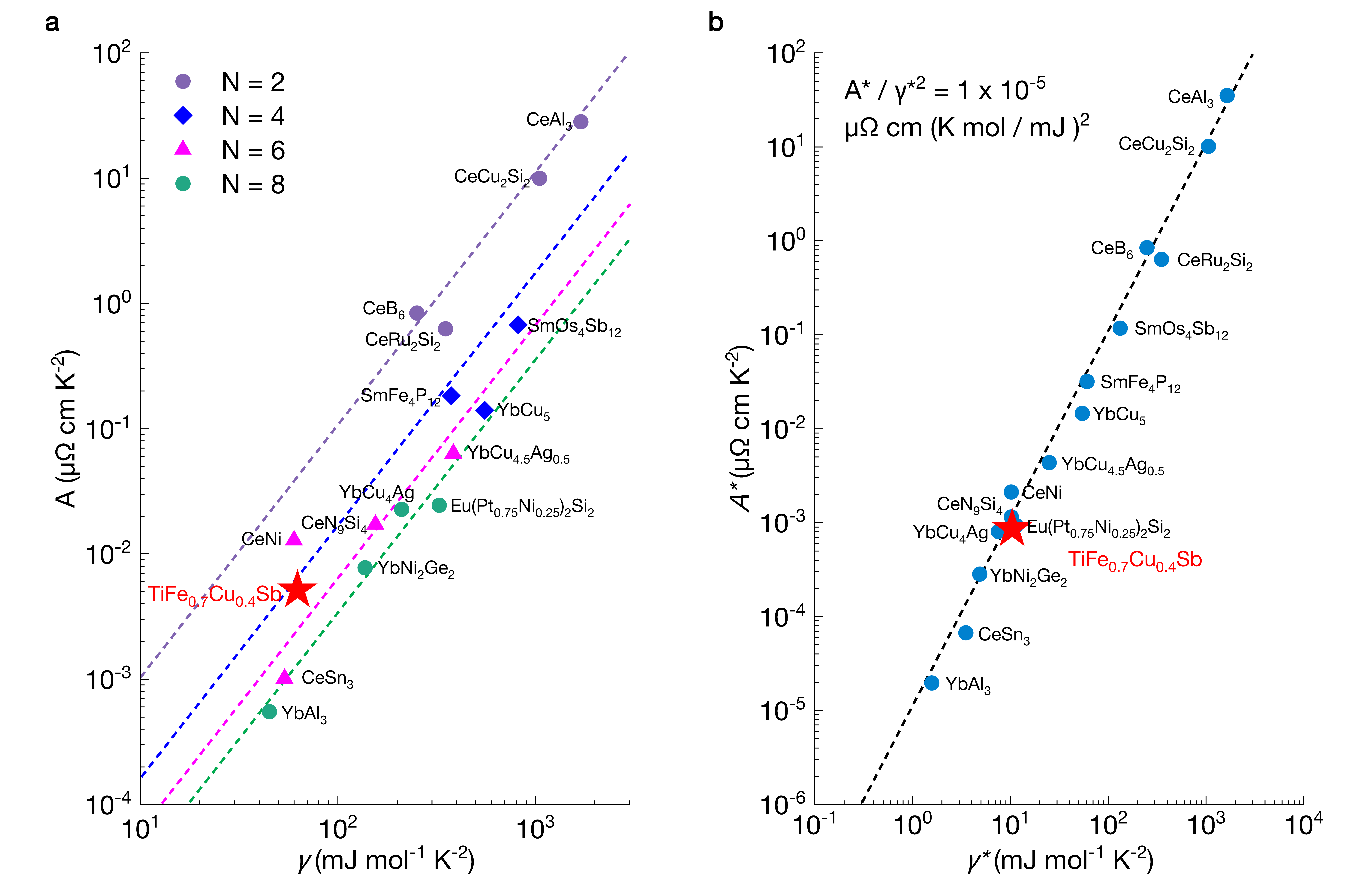


**Fig. S10 | Kadowaki-Woods relation in TiFe_0.7_Cu_0.4_Sb. a,** The Sommerfeld coefficient $\gamma$ obtained at $\mu_{0}H=$ 0 T is 62.55 $\text{mJ }\text{mole-Fe}^{\text{-1}}\text{ }\text{K}^{\text{-2}}$. The coefficient $A$ is obtained by fitting the resistivity at $\mu_{0}H=$ 0 T to $\rho(T)=\rho_{0}+AT^{2}$ below $T=$ 0.30 K, yielding $A=$ 0.51489$\pm$0.00211 $\times{10}^{-2} \Omega cm K^{-2}$. The position of TiFe_0.7_Cu_0.4_Sb in the $A$- $\gamma$ diagram is plotted in (**a**), along with the data of other heavy-fermion materials as classified by their spin degeneracy *N*. The dashed lines are the prediction from the orbitally degenerate periodic-Anderson model ^53,54^. **b**, In TiFe_0.7_Cu_0.4_Sb, $N=$ 4 due to the two-fold degeneracy of $e_{g}$ orbital. When the spin degeneracy is taken into consideration, $A^{*}=A/\frac{1}{2}N(N-1)$, and $\gamma^{*}=\gamma/\frac{1}{2}N(N-1)$ could be defined. The Kadowaki-Woods quantity, as defined by $A^{*}/\gamma^{*2}$ has been theoretically derived to be a constant value of 1$\times$10^-5^ $\mu\Omega cm\left( K mol / mJ \right)^{2}$ (black dashed line in **b**) ^55^, which is the found hold in many heavy-fermion materials. We found TiFe_0.7_Cu_0.4_Sb also following this relation. The experimental data are collected from Ref. ^54,56-59^.

**Sec. 10: Fitting of the magnetic** **Grüneisen parameter**

In the main text, we fit the magnetic Grüneisen parameter using $\Gamma_{B}\left( \mu_{0}H \right)=A/\left( \mu_{0}H-\mu_{0}H_{c} \right)^{vz}$ at $\mu_{0}H>$ 0.14 T regime by fixing $\mu_{0}H_{c}=$ 0.13 T as obtained from the specific heat measurements. In Fig. S11, we have fitted the data point to the formula in different conditions.

We also fitted the $\Gamma_{B}\left( \mu_{0}H \right)$ by setting all parameters free, as shown in Fig. S11b, but the obtain parameters, especially $\mu_{0}H_{c}$ has a very large uncertainty, ranging from -0.108 T to 0.062 T that covers the unphysical negative regimes. For AFM or FM materials possessing Hertz-Millis type of QCPs, the critical exponent $vz$ is 1 or 3/2, respectively. In Fig. S11c and d, we fitted the $\Gamma_{B}\left( \mu_{0}H \right)$ by fixing $vz=$1 and 3/2, and the obtained critical magnetic field $\mu_{0}H_{c}$ is also a unphysical negative value, which indicates the QCP as observed here is not conform to the Hertz-Millis type of QCPs as in AFM or FM quantum critical materials.

Because of the large noise in our measurements due to the small magnetic moments and for a fair report, we fixed $\mu_{0}H_{c}=$ 0.13 T in the fitting as shown in the main text (Fig. 2e). A complete characterization of the critical behaviours requires future experimental as well as theoretical work to refine and understand the results.


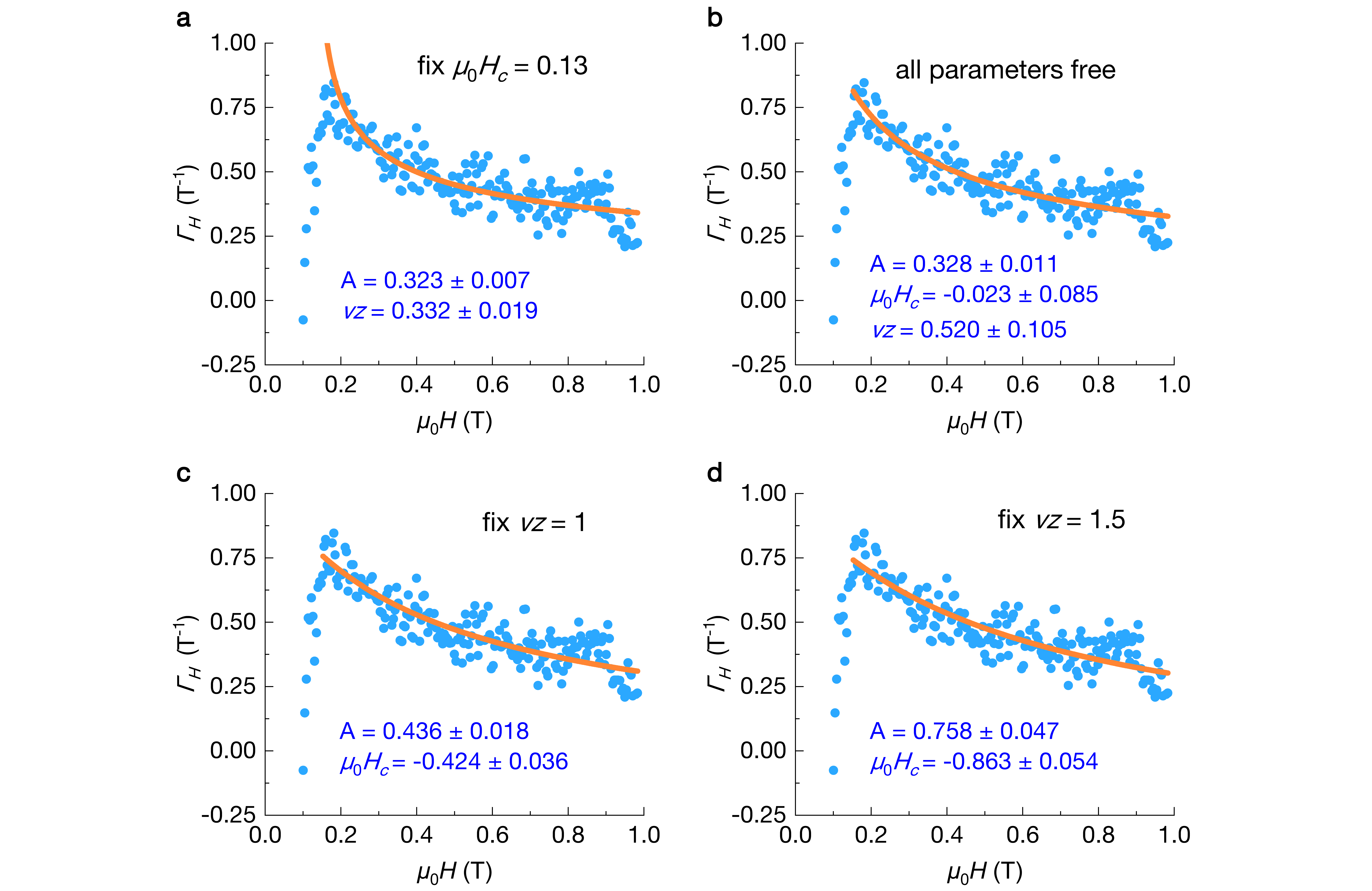


**Fig. S11 | Fitting of the magnetic Grüneisen parameter. a**, The magnetic Grüneisen parameter, $\Gamma_{B}\left( \mu_{0}H \right)$, is fitted by fixing $\mu_{0}H_{c}=$ 0.13 T, with the fitted values indicated in blue. **b**, A direct fit without fixed parameters shows large uncertainty in $\mu_{0}H_{c}$. **c**, $\Gamma_{B}\left( \mu_{0}H \right)$ is fitted by fixing the critical exponent $vz=$ 1. **d**, The same fitting process as in (**c)** but with $vz=$ 1.5. The negative critical field values suggest the observed quantum critical point does not conform to the Hertz-Millis type in antiferromagnetic or ferromagnetic systems.

**Sec. 11: Temperature dependence of the magnetic Grüneisen parameter**

To further support the existence of a magnetic-field-driven QCP, we have analyzed the temperature dependence of the magnetic Grüneisen parameter $\Gamma_{H}(T)$ at several magnetic fields via the thermodynamic relation $\Gamma_{H}=-\left( {dM}/{dT} \right)/C_{el}$, where $M$ is the dc magnetization and $C_{el}$ is the electronic specific heat. In Fig. S12, we present (a) $-\left( {dM}/{dT} \right)/T$, (b) $C_{el}(T)/T$, and (c) their ratio $\Gamma_{H}(T)$ at $T>$ 0.40 K region. At $\mu_{0}H_{c}=$ 0.13 T, both $-\left( {dM}/{dT} \right)/T$ and $C_{el}(T)/T$ increase upon cooling, and the stronger divergence in $-\left( {dM}/{dT} \right)/T$ leads to a divergent $\Gamma_{H}(T)$. Thus, within our accessible temperature range of $T>$ 0.40 K, the magnetic Grüneisen parameter $\Gamma_{H}(T)$ diverges at $\mu_{0}H_{c}=$ 0.13 T, supporting the presence of a field-induced QCP in TiFe_0.7_Cu_0.4_Sb. At a slightly higher field of $\mu_{0}H=$ 0.20 T, $\Gamma_{H}(T)$ also exhibits a divergence above 0.40 K. A possible explanation is that $\Gamma_{H}(T)$ would eventually saturate below 0.40 K (which is lower than the minimum temperature we can reach in measuring dc magnetization).

**

**

**Fig. S12 | Temperature dependence of the magnetic Grüneisen parameter at various magnetic fields.** **a**, Temperature derivative of the magnetization as $-\left( {dM}/{dT} \right)/T$, **b**, electronic specific heat as $C_{el}/T$, and **c**, magnetic Grüneisen parameter $\Gamma_{H}$ versus temperature $T$ on a logarithmic scale for polycrystalline TiFe_0.7_Cu_0.4_Sb in various magnetic fields.

**Sec. 12: Estimation of the Kondo temperature**


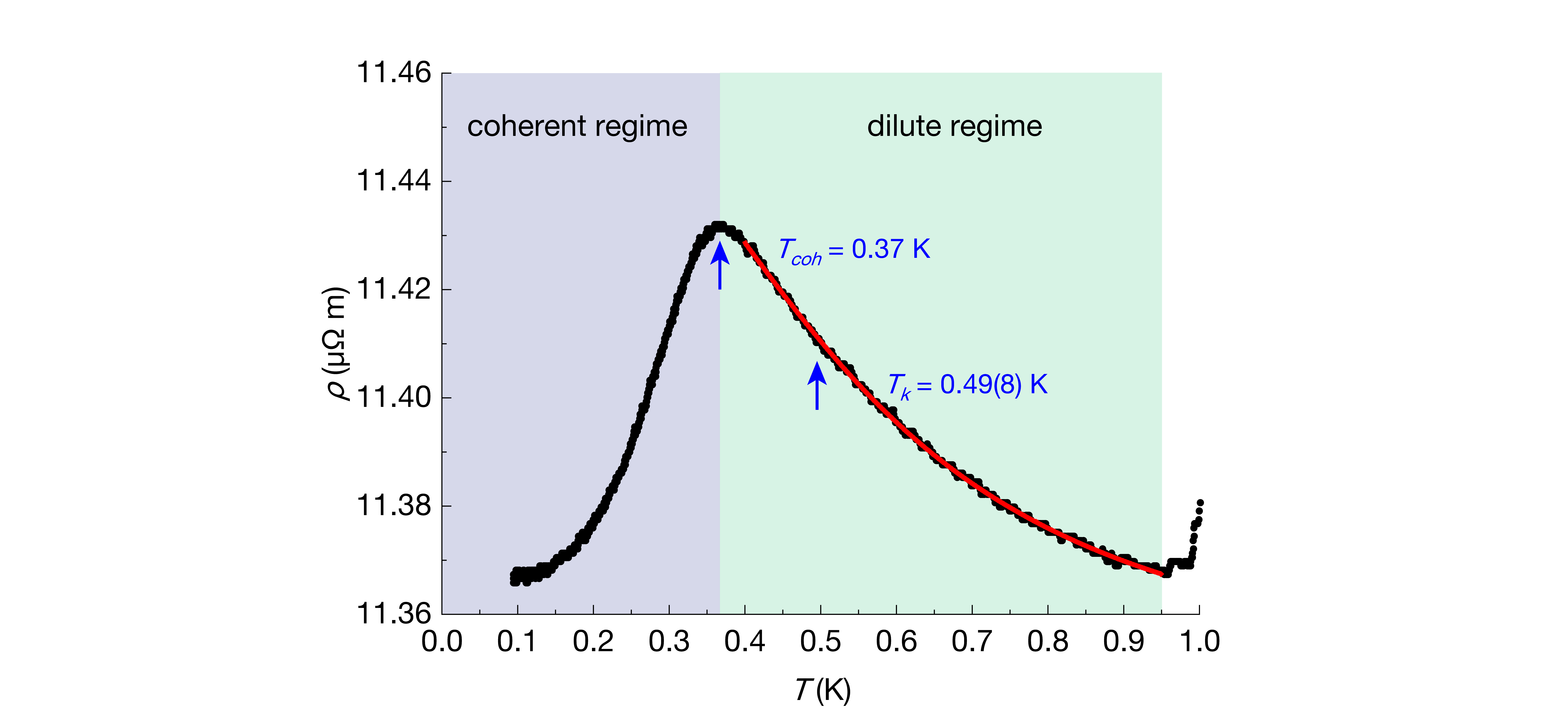


**Fig. S13: Estimation of the Kondo temperature.** The resistivity $\rho(T)$ at $\mu_{o}H=$ 0 T of TiFe_0.7_Cu_0.4_Sb has the similar behaviour to other dilute Kondo disordered systems. Above 0.95 K, it displays metallic behaviour (see Fig. S5a), followed by a resistivity minimum at 0.95 K. Below this temperature, the resistivity increases with cooling (following a $-lnT$ dependence), indicating dilute single-ion Kondo scattering^60^. As the temperature is further decreased, the coherent Kondo interaction develops, featuring that $\rho(T)$ decreases with cooling, and the resistivity maximum can be intuitively defined as the coherent temperature $T_{coh}$ (see details in main text). To estimate the Kondo temperature $T_{K}$, we use the Hamann’s expression $\rho\left( T \right)=\rho_{0}+C\left\{ 1-{\ln\left( \frac{T}{T_{K}} \right)}/{\sqrt{\ln^{2}\left( \frac{T}{T_{K}} \right)+D}} \right\}$ to fit $\rho(T)$ in the dilute regime^61^. The red line is the fit in the temperature region from 0.40 K to 0.95 K, and the obtained Kondo temperature $T_{K}$ is 0.498 $\pm$ 0.004 K.

**Sec. 13: Crossover between coherent and dilute regimes**

As elaborated in the main text, the high-temperature electrical resistivity $\rho(T)$ is typical of uncorrelated, paramagnetic local moments in the presence of single-ion impurity Kondo hybridization with the conduction electrons, which is responsible for the negative slope. At temperatures below a crossover marked by maximal resistivity $\rho(T)$, the Kondo hybridization yields coherent electronic bands, resulting in a metallic temperature-dependence of the resistivity $\rho(T)$ due to the emerging of coherent Kondo interactions.

From the resistivity measurements, we have determined the regimes separating coherent and dilute Kondo regimes marked by the coherent temperature scale $T_{coh}$, which is determined as the maximum point at the resistivity $\rho(T)$, as shown in Fig. S14a as red arrows.

Moreover, when the system transitions from the dilute Kondo impurity scattering to Kondo coherent scattering, the electronic behaviours are changed, which affects the magnetoresistivity as well. From the magnetoresistivity $\rho(\mu_{0}H)$ measurmenets, we observed the $\rho(\mu_{0}H)$ has a crossover from larger slope to a small slope with increasing the magnetic fields $\mu_{0}H$, while the coherent magnetic field scale ${\mu_{0}H}_{coh}$ is denoted by the blue arrows in Fig. S14b.

In Fig. S14c, we plotted $T_{coh}$ and ${\mu_{0}H}_{coh}$, which coincides together separating the Kondo coherent and dilute regimes. The phase diagram reveals that increasing magnetic fields suppresses the Kondo coherent regime. This observation aligns with the Kondo breakdown scenario, where applying magnetic fields enhances Kondo screening, effectively reducing the number of active local moments as they form composite fermions with conduction electrons. Consequently, the coherent interactions between magnetic moments are gradually suppressed as more magnetic moments become screened.


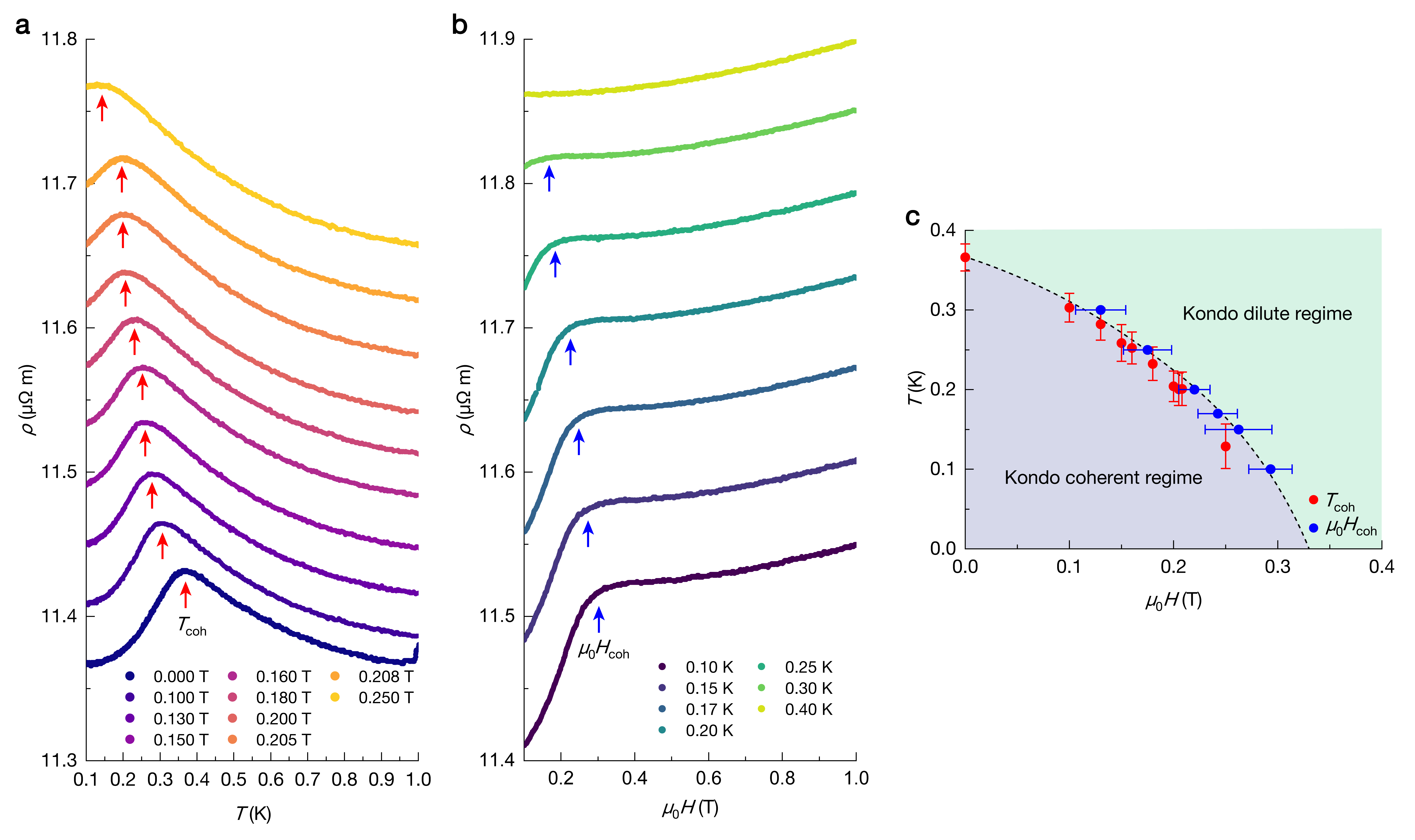


**Fig. S14: Phase diagram of Kondo coherent and Kondo dilute regimes. a**, the resistivity $\rho(T)$ measurements, and the temperature scale $T_{coh}$ is marked by the red arrows. **b**, the magnetoresistivity measurements, and the magnetic field scale ${\mu_{0}H}_{coh}$ is marked by blue arrows. **c**, the phase diagram which separates the Kondo coherent and dilute regimes.

**Sec. 14: Extracting the Hall resistivity** $\boldsymbol{\rho}_{\boldsymbol{H}}$ **from the raw data**


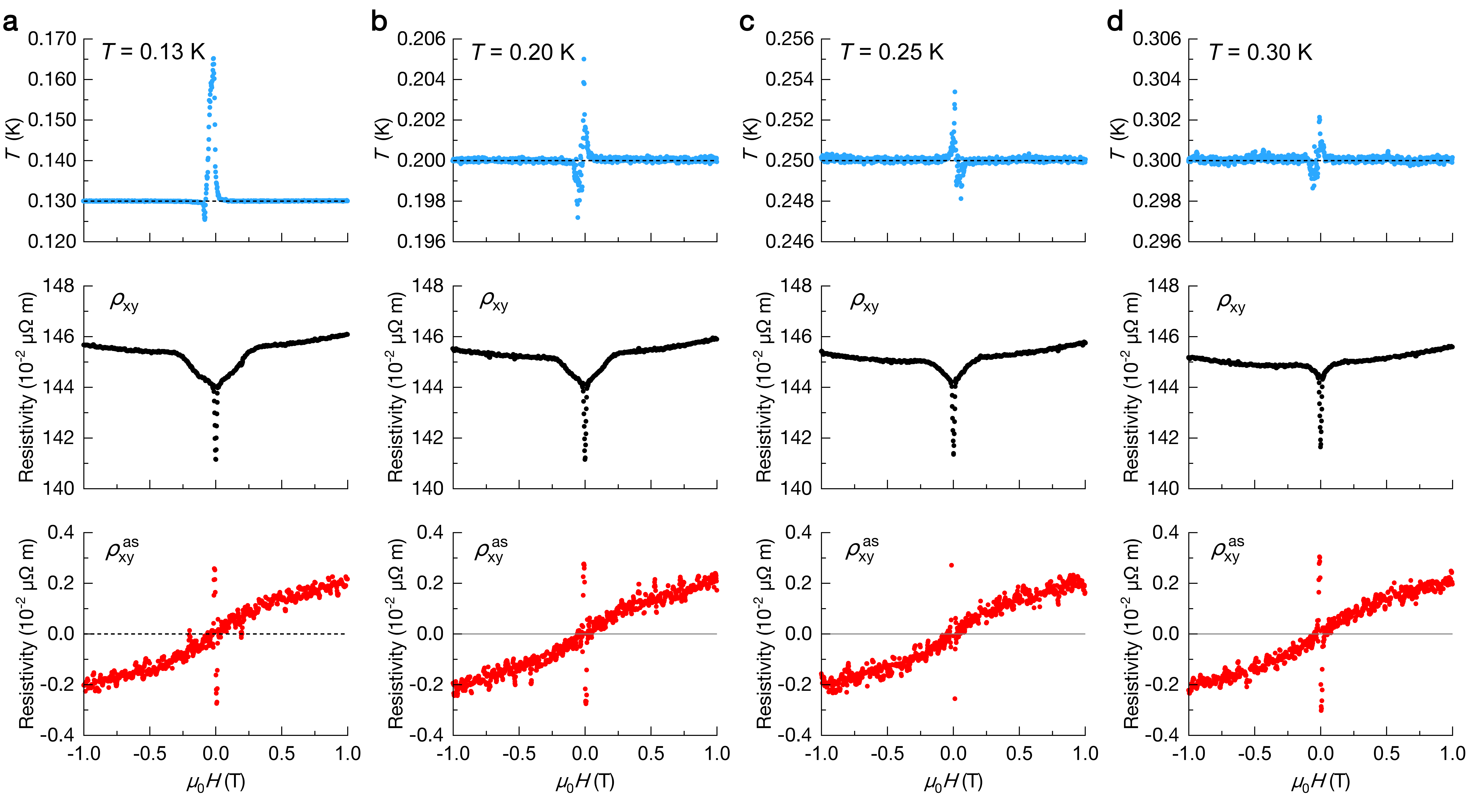


**Fig. S15 | Anti-symmetrization of Hall resistivity.** The raw Hall resistivity $\rho_{xy}$ contains contributions from magnetoresistivity. To obtain the intrinsic Hall resistivity, we calculate the anti-symmetrized Hall resistivity as $\rho_{xy}^{as}(\mu_{0}H)=\left[ \left( \rho_{xy}\left( \mu_{0}H \right)-\rho_{xy}\left( -\mu_{0}H \right) \right) \right]/2$, where $\rho_{xy}\left( \mu_{0}H \right)$ is the measured raw Hall resistivity, and $\rho_{xy}^{as}(\mu_{0}H)$ is taken as the real Hall resistivity, denoted as $\rho_{H}$. The first row shows the recorded temperature during the measurements. When the magnetic field $\mu_{0}H$ is flipped from negative to positive values, the temperature becomes unstable. The second row displays the raw Hall resistivity $\rho_{xy}$, which includes significant contributions from magnetoresistivity. The third row presents the anti-symmetrized resistivity $\rho_{xy}^{as}$. Due to temperature instability during magnetic field flips, in the main text we only consider the regime where the magnetic field $\mu_{0}H>$ 0.1 T. To estimate the anomalous Hall resistivity $C_{s}M(\mu_{0}H)$, we used the magnetization data at 0.4 K shown in Fig. S5b. The coefficient $C_{s}$ was obtained from a linear fit of the anti-symmetrized Hall resistivity, $\rho_{xy}^{as}\left( \mu_{0}H \right)$, at 0.30 K using the relation $\rho_{xy}^{as}\left( \mu_{0}H \right)=\rho_{H}\left( \mu_{0}H \right)+C_{s}M(\mu_{0}H)$ in the high-field range 0.8 to 1.0 T, where $M(\mu_{0}H)$ remains a constant at approximately 0.04 $\mu_{B}$. From this analysis, we estimate that the anomalous Hall resistivity contributes about 10% of the total anti-symmetrized Hall signal at $\mu_{0}H=$ 0.30 T. Therefore, in the main text we directly denote the anti-symmetrized $\rho_{xy}^{as}\left( \mu_{0}H \right)$ as the ordinary Hall resistivity $\rho_{H}(\mu_{0}H)$.

**Sec. 15: Quantitative analysis of the crossover functions in Hall measurements**

**
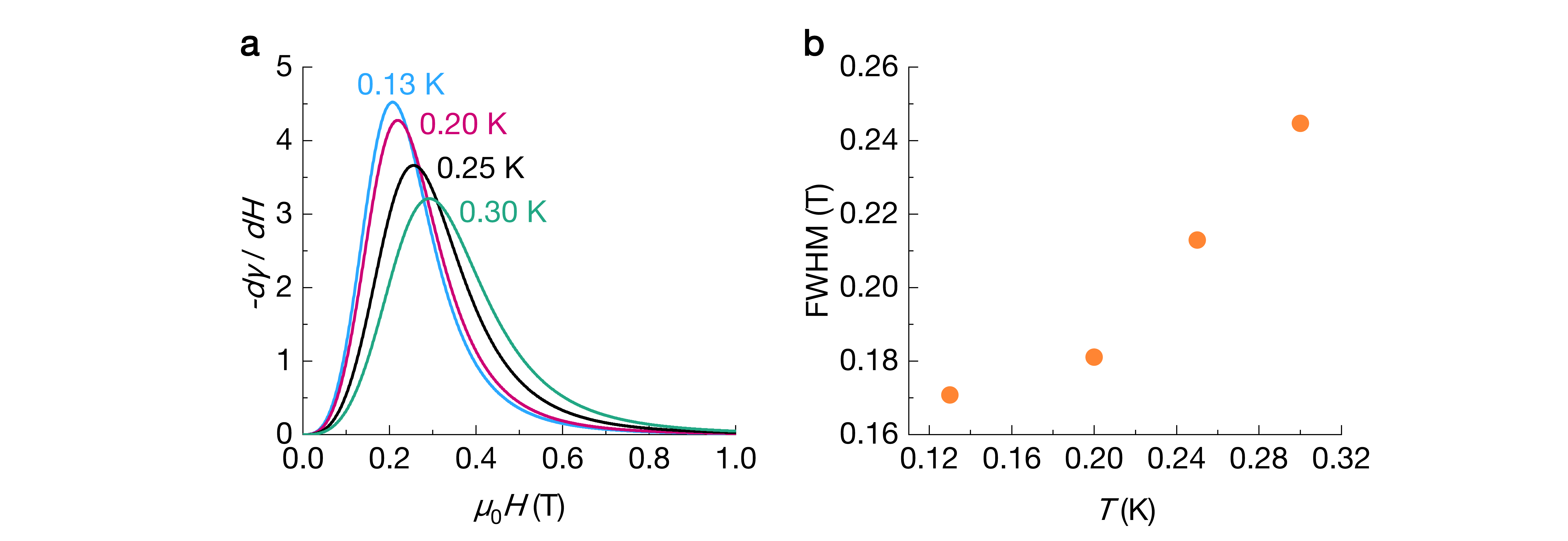
**

**Fig. S16 | Quantitative analysis of the crossover function** $\boldsymbol{\gamma(}\boldsymbol{\mu}_{\boldsymbol{0}}\boldsymbol{H)}$**.** As described in the main text, we used a crossover function $\gamma(\mu_{0}H$) to fit the Hall resistivity. In panel (**a)**, we plot the field derivative of the crossover function, which can be interpreted as the rate of change of the effective carrier concentration. As the temperature approaches zero, the crossover function is expected to diverge at the QCP. Panel (**b**) shows the full width at half maximum (FWHM), indicating the width of the crossover. With decreasing temperature, the FWHM decreases, consistent with the sharpening expected for quantum criticality, as observed in other materials exhibiting a QCP. Moreover, since the Hall effect was measured at temperatures on the same scale as the Kondo temperature, unlike in YbRh_2_Si_2_ where measurements extended to three orders of magnitude below $T_{K}$, it is natural to expect the FWHM to extrapolate to non-zero in our case^62,63^. The observed tendency of the FWHM to saturate at finite values is therefore a natural consequence of the limited temperature range relative to and maybe further broadened by intrinsic disorder. Nevertheless, the clear narrowing of the crossover with decreasing temperature still provides strong evidence for the underlying Kondo-breakdown QPTs.

**Sec. 16: Effective Hamiltonian of TiFe_x_Cu_2x-1_Sb**

Due to the complex structure of TiFe_x_Cu_2x-1_Sb, its effective Hamiltonian is composed of following terms

$$\begin{aligned} H=H_{c}+H_{d}+H_{I}\#\left( S6 \right) \end{aligned}$$

where $H_{c}$ comes from conduction electrons, $H_{d}$ is from localized electrons hosting local moments, $H_{I}$ is the hybridization between conduction electron and localized electrons.

The conduction electrons in TiFe_x_Cu_2x-1_Sb are contributed by the partially itinerant 3*d*-electrons from Ti atoms, and the itinerant 5*p*-electrons from Sb atoms (copper is used to modify the electrons concentration).

$$\begin{aligned} H_{c}=\sum_{ij\sigma} t_{ij}^{1}a_{i\sigma}^{\dagger}a_{j\sigma}+\sum_{ij\sigma} t_{ij}^{2}b_{i\sigma}^{\dagger}b_{j\sigma}+\sum_{ij\sigma} t_{ij}^{0}a_{i\sigma}^{\dagger}b_{j\sigma}+h.c.\#\left( S7 \right) \end{aligned}$$

where $a_{i\sigma}^{\dagger}$ and $b_{j\sigma}$ are the creation operator of 3*d*-electrons from Ti atoms and 5*p*-electrons from Sb atoms, respectively; $\sigma$ denotes the spin index, $\uparrow$ and $\downarrow$; $t_{ij}^{1}$, $t_{ij}^{2}$, $t_{ij}^{0}$ is the hopping amplitude between Ti-Ti sites, Sb-Sb sites and Ti-Sb sites. h.c. stands for Hermitian conjugate. Generally, Ti and Sb are different sites with different types of electrons; however, to our purpose here which only acquire their itinerant nature (the correlation effect of Ti *d*-electrons and the small magnetic moments are thus neglected), we may consider *d*-electrons from Ti and *p*-electrons from Sb as the “same type” $a_{i\sigma}^{\dagger}\approx b_{i\sigma}^{\dagger}=c_{i\sigma}^{\dagger}$ with different “orbital” flavors, $c_{i\sigma}^{\dagger}\to c_{i\sigma\alpha}^{\dagger}$ ($\alpha$ refers to flavor index). Eq. S7 can be rewritten as

$$\begin{aligned} H_{c}=\sum_{ij\sigma\alpha} t_{ij}c_{i\sigma\alpha}^{\dagger}c_{j\sigma\alpha}\#\left( S8 \right) \end{aligned}$$

where $t_{ij}$ is the hopping amplitude between different lattice and basis sites.

The localized electrons which host local moments are mostly originated from Fe atoms. The three-fold degenerate $t_{2g}$ orbitals of 3*d*-electrons in Fe are full occupied, whereas the two-fold degenerate $e_{g}$ orbital is only occupied by one electron, and thus the local spins have a degeneracy $N=$ 4. The Hamiltonian from Fe *d*-electrons is composed of

$$\begin{aligned} H_{d}=\sum_{i\sigma\alpha} E_{d}d_{i\sigma\alpha}^{\dagger}d_{i\sigma\alpha}+U\sum_{i\sigma\alpha} d_{i\sigma\alpha}^{\dagger}d_{i\sigma\alpha}d_{i\bar{\sigma}\alpha}^{\dagger}d_{i\bar{\sigma}\alpha}+\sum_{ij} \tilde{J}_{ij}\boldsymbol{S}_{i}\cdot\boldsymbol{S}_{j}\#\left( S9 \right) \end{aligned}$$

where the first two terms are the interaction from single Fe sites, and the RKKY interaction between Fe sites is captured by the third term. Here, $d_{i\sigma\alpha}^{\dagger}$ is the creation operator of *d*-electrons from Fe atoms. $E_{d}$ is the energy of single Fe state with $e_{g}$-orbital filled of one electron, $U$ is the correlation energy of Fe $e_{g}$-orbital. $\boldsymbol{S}_{i}$ is the spin operator of local moment, and $\tilde{J}_{ij}$ is the exchange interaction between local moments. $\bar{\sigma}=-\sigma$.

The unique crystal structure of TiFe_0.7_Cu_0.4_Sb is that Fe atoms are randomly distributed (see Fig. S17). As results from this, (a) the local moments $\boldsymbol{S}_{i}$ are fluctuating from sites to sites, i.e., $\boldsymbol{S}_{i}=a_{i}\boldsymbol{S}$, where $a_{i}$ are random numbers that $a_{i}\in\left[ 0, 1 \right]$; and (b) the exchange interaction between local moments $\tilde{J}_{ij}$ are randomly distributed. For easier theoretical analysis, we may incorporate the fluctuating nature of $\boldsymbol{S}_{i}$ (i.e., $a_{i}$) into the random distributed $\tilde{J}_{ij}$ by redefining that $J_{ij}\equiv a_{i}a_{j}\tilde{J}_{ij}$. Then, the local moments $\boldsymbol{S}_{i}$ in eq. (S9) has regular behaviours as in periodic Kondo lattice, whereas the matrix elements of $J_{ij}$, despite having many zero elements, are still random as to counter not only the fluctuation of local moments but also the randomly distributed exchange interactions.

Most importantly, there is a hybridization term between conduction electrons and localized *d*-electrons, as

$$\begin{aligned} H_{I}=\sum_{ij\sigma\alpha} \left( V_{ij\sigma\alpha}d_{i\sigma\alpha}^{\dagger}c_{j\sigma\alpha}+V_{ij\sigma\alpha}^{*}c_{j\sigma\alpha}^{\dagger}d_{i\sigma\alpha} \right)\#\left( S10 \right) \end{aligned}$$

where $V_{ij\sigma\alpha}$ is the hybridization constants.

Therefore, the total Hamiltonian of TiFe_x_Cu_2x-1_Sb is then

$$H=\sum_{ij\sigma\alpha} t_{ij}c_{i\sigma\alpha}^{\dagger}c_{j\sigma\alpha}+\sum_{ij} J_{ij}\boldsymbol{S}_{i}\boldsymbol{S}_{j}$$

$$\begin{aligned} +\sum_{i\sigma\alpha} E_{d}d_{i\sigma\alpha}^{\dagger}d_{i\sigma\alpha}+U\sum_{i\sigma\alpha} d_{i\sigma\alpha}^{\dagger}d_{i\sigma\alpha}d_{i\bar{\sigma}\alpha}^{\dagger}d_{i\bar{\sigma}\alpha}+\sum_{ij\sigma\alpha} \left( V_{ij\sigma\alpha}d_{i\sigma\alpha}^{\dagger}c_{j\sigma\alpha}+V_{ij\sigma\alpha}^{*}c_{j\sigma\alpha}^{\dagger}d_{i\sigma\alpha} \right)\#\left( S11 \right) \end{aligned}$$

where the three terms in the second line composes of an Anderson impurity model. Taking the hybridization term involving $V_{ij\sigma\alpha}$ and $V_{ij\sigma\alpha}^{*}$ as perturbations, the three occupied states which can span the space are $\left. |d^{1} \right\rangle$, $\left. |d^{0} \right\rangle$ and $\left. |d^{2} \right\rangle$, with energies of $E_{f}$, $E_{f}+E_{d}$, and $E_{f}+2E_{d}+U$.

At first, we can ignore the double occupied $\left. |d^{2} \right\rangle$ state. Compared with $\left. |d^{1} \right\rangle$ or $\left. |d^{0} \right\rangle$ state, the energy level of $\left. |d^{2} \right\rangle$ state is very large due to the presence of $U$ that typically around 1 eV (where $E_{d}$ is around $-$0.1 eV). So, the space is only spanned by state $\left. |d^{1} \right\rangle$ and $\left. |d^{0} \right\rangle$. Introducing the Hubbard operator $d_{\sigma}^{\dagger}=X_{\sigma0}=\left. |d^{1},\sigma\right\rangle\left\langle d^{0}| \right.$; $d_{\sigma}=X_{0\sigma}=\left. |d^{0} \right\rangle\left\langle d^{1},\sigma| \right.$; and $d_{\sigma}^{\dagger}d_{\sigma}=X_{\sigma\sigma}=\left. |d^{1},\sigma\right\rangle\left\langle d^{1},\sigma| \right.$, eq. S11 is renormalized as:

$$H=\sum_{ij\sigma\alpha} t_{ij}c_{i\sigma\alpha}^{\dagger}c_{j\sigma\alpha}+\sum_{ij} J_{ij}\boldsymbol{S}_{i}\boldsymbol{S}_{j}$$

$$\begin{aligned} +E_{d}\sum_{i\sigma\alpha} X_{\sigma\sigma}^{i\alpha}+\sum_{ij\sigma\alpha} \left( V_{ij\sigma\alpha}X_{\sigma0}^{i\alpha}c_{j\sigma\alpha}+V_{ij\sigma\alpha}^{*}c_{j\sigma\alpha}^{\dagger}X_{0\sigma}^{i\alpha} \right)\#\left( S12 \right) \end{aligned}$$

where the double occupied states (the term relating to $U$) is projected out.

In the present research, we explore the QCP physics at $T\to$ 0 limit, and hence the low-energy Hilbert space is only involving the $\left. |d^{1} \right\rangle$ state. The residual $\left. |d^{1} \right\rangle$ state still interact with the surrounding conduction sea for virtual charge fluctuations via the process: $\left. |d^{1},\sigma\right\rangle+e_{\bar{\sigma}}⟷e_{\bar{\sigma}}+e_{\sigma}⟷\left. |d^{1},\bar{\sigma} \right\rangle+e_{\sigma}$ with energy $-E_{d}$ ($e_{\sigma}$, conduction electron with spin $\sigma$), which is the only process that we take into account. From second-order perturbation theory, this virtual charge fluctuation will lower the energy by an amount of order $\Delta E=-J_{K}$, where $J_{K}=V^{2}/E_{d}$. The reduction in the energy constitutes an effective antiferromagnetic interaction between the conduction electrons and the local moments. Introducing the operator $\sigma\left( 0 \right)=\sum_{i,j} c_{i\alpha}^{\dagger}\sigma_{\alpha\beta}c_{j\beta}$, which measures the electron spin at the origin ($\sigma_{\alpha\beta}$ is the Pauli matrix), the effective interaction between the conduction electrons and $d$-electrons will have the form $H_{eff}=J_{K}S\cdot\sigma\left( 0 \right)$. Thus, eq. S12 is rewritten as:

$$\begin{aligned} H=\sum_{ij\sigma\alpha} t_{ij}c_{i\sigma\alpha}^{\dagger}c_{j\sigma\alpha}+\sum_{i<j} J_{ij}\boldsymbol{S}_{i}\cdot\boldsymbol{S}_{j}+J_{K}\sum_{i\sigma\sigma^{'}\alpha} \boldsymbol{S}_{i}\cdot\left( c_{i\sigma\alpha}^{\dagger}\boldsymbol{\sigma}_{\sigma\sigma^{'}}c_{i\sigma^{'}\alpha} \right)\#\left( S13 \right) \end{aligned}$$

which is the so called random-exchange Heisenberg-Kondo Hamiltonian.

**
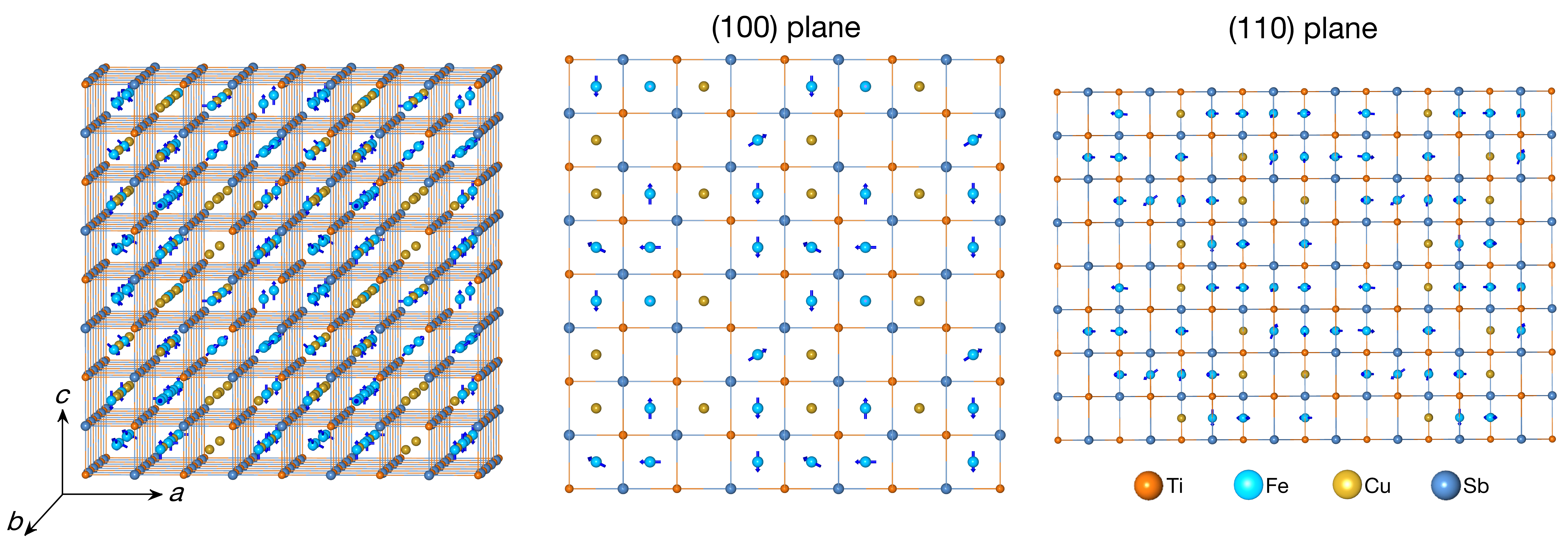
**

**Fig. S17 | Supercell crystal structure of TiFe_0.7_Cu_0.4_Sb.** Left, The Ti and Sb atoms form the skeleton of a face-centered cubic structure, while Fe/Cu atoms partially occupy 4*c* and 4*d* Wyckoff sites at random. The local magnetic moments, primarily located on the Fe atoms, are randomly distributed, with nearest neighboring antiferromagnetic interactions. The middle and right panels show views from (100) plane and (110) plane, respectively. The arrows on the Fe atoms are intended to indicate the possible presence of magnetic moments but do not represent the magnitude of these moments.

**Sec. 17: ac and dc magnetic susceptibility at finite magnetic fields**





**Fig. S18 | ac susceptibility** $\boldsymbol{\chi}^{\boldsymbol{'}}\boldsymbol{(T)}$ **at finite magnetic fields.** With increasing the magnetic fields, the peaks in $\chi^{'}(T)$ are gradually suppressed and the peaks height do not monotonically decrease with increasing the frequency as shown in **a**, **b** and **c**, indicating the cluster spin-glass phase is gradually diminishing and goes to the heavy-fermion liquid phase. Due to the suppression of the peaks, we cannot determine the position of $T_{f}$ at finite magnetic fields preciously, as reflected in the gradually increased error bars from **d** to **h**. We also tried to calculate the $\delta T_{f}$, as shown in **i**, which also shows significant error bars at large magnetic fields. Thus, a quantitative description of the suppression of cluster spin-glass phase cannot be given, but a qualitative description that the cluster spin-glass phase is suppressed with increasing fields can certain be concluded.

**
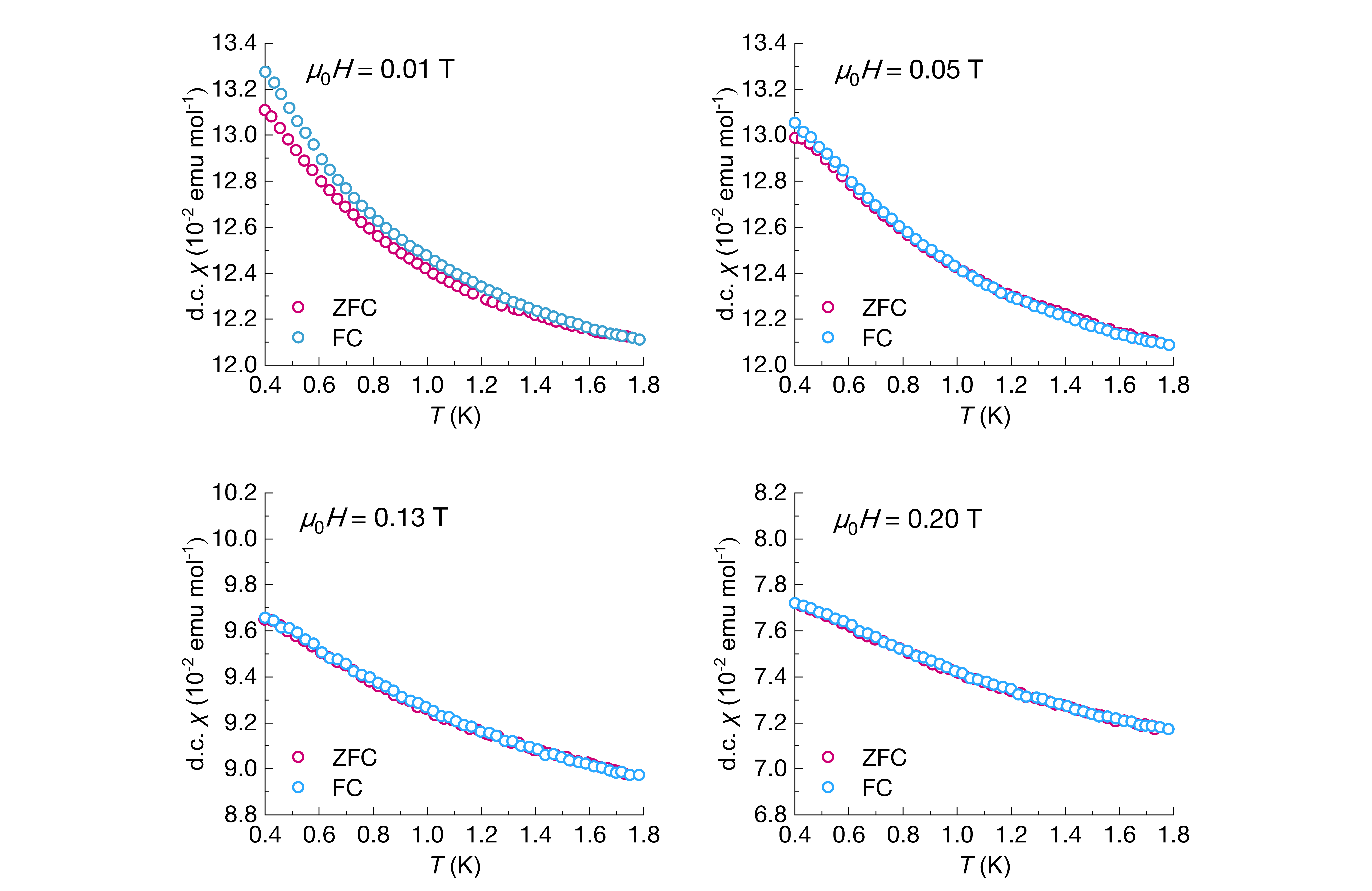
**

**Fig. S19 | dc susceptibility** $\boldsymbol{\chi(T)}$ **at finite magnetic fields.** As the magnetic field increases, we observe that the FC and ZFC curves gradually level off, and the difference between them diminishes at 0.13 T. These features indicate that the cluster spin-glass phase at $T<$ 0.4 K is progressively suppressed as the magnetic field strengthens.

**Sec. 18: Schematic of global phase diagram**


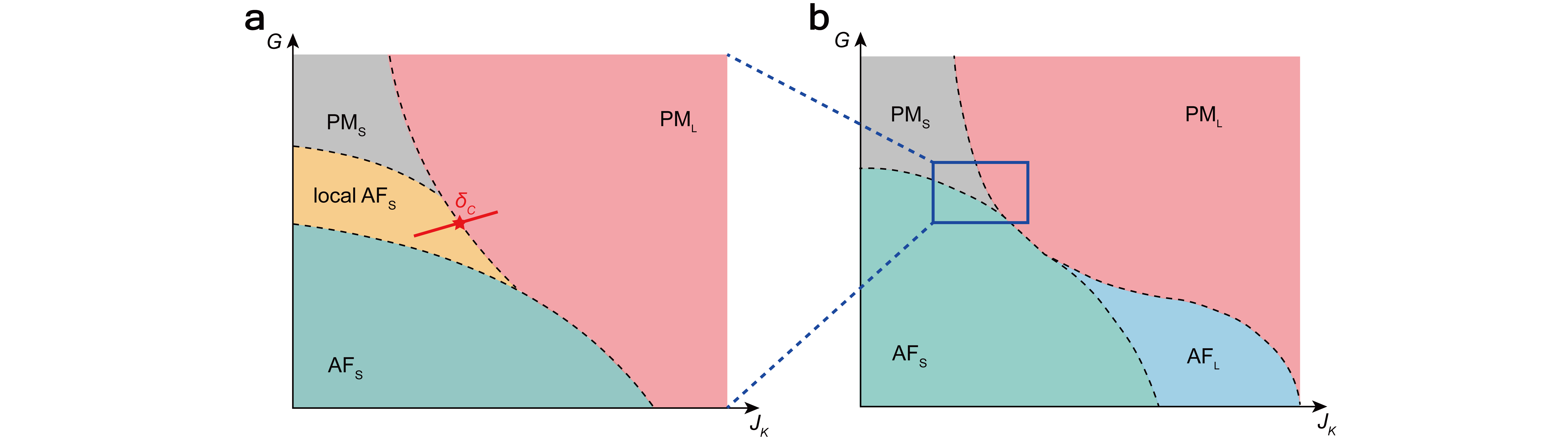


**Fig. S20: Schematic of global phase diagram.** **a**, The modified phase diagram at the area denoted by blue rectangle in (**b)**, which is suitable for TiFe_0.7_Cu_0.4_Sb. $J_{K}$ tunes the Kondo coupling and $G$ varies the degree of magnetic frustration. PM and AF stand for paramagnetic and antiferromagnetic phases, and the subscripts L and S denote the large and small Fermi surface. Since the cluster spin glass contains local antiferromagnetic pairs without long-range magnetic order, generally speaking, it is not an AF or PM states, but a state in between denoted by local AF_s_. The red line denotes the path for phase transition, and $\delta_{c}$ is the point of Kondo breakdown QCP, where $\delta$ is the turning parameter such as magnetic field here. **b**, Normal global phase diagram for heavy-fermion metals ^64,65^.

**Table S1: Parameters for substruction of the phonon** $\boldsymbol{C}_{\boldsymbol{ph}}$**, nuclear** $\boldsymbol{C}_{\boldsymbol{nuc}}$ **and magnetic Schottky contribution** $\boldsymbol{C}_{\boldsymbol{sch}}$ **to the total specific heat.** For phonon contributions ($C_{ph}=\beta T^{3}$), we only fitted the data at zero field, and the same $\beta$ was employed for finite fields. For nuclear contributions ($C_{nuc}=A/T^{2}$), we fitted the data at all magnetic fields at $T<$ 0.1 K region. For magnetic Schottky contributions, $n$ and $\Delta$ are determined by the peak height and position of $C_{m}$, respectively. Note that at these magnetic fields of 0.025, 0.075, 0.140 and 0.250 T, specific heat is only measured below 0.15 K. N/A: not applicable.

| $\boldsymbol{\mu}_{\boldsymbol{0}}\boldsymbol{H}$ (T) | $\boldsymbol{\beta}$ (J mol^-1^ K^2^) | $\boldsymbol{A}$ (10^-5^J mol^-1^ K) | $\boldsymbol{n}$ (%) | $\boldsymbol{\Delta}$ (K) |
| --- | --- | --- | --- | --- |
| 0 | 0.00105$\pm$0.00006 | 1.93488$\pm$0.02384 | 0.28 | 1.16 |
| 0.025 | / | 1.90843$\pm$0.02659 | N/A | N/A |
| 0.050 | / | 1.91530$\pm$0.03160 | 0.27 | 1.20 |
| 0.075 | / | 1.84519$\pm$0.03298 | N/A | N/A |
| 0.100 | / | 1.86741$\pm$0.05257 | 0.26 | 1.24 |
| 0.125 | / | 1.86359$\pm$0.03050 | 0.26 | 1.26 |
| 0.130 | / | 1.83464$\pm$0.02310 | 0.27 | 1.27 |
| 0.140 | / | 1.87062$\pm$0.02728 | N/A | N/A |
| 0.150 | / | 1.89023$\pm$0.03213 | 0.26 | 1.31 |
| 0.175 | / | 1.77218$\pm$0.02908 | 0.26 | 1.36 |
| 0.200 | / | 1.81643$\pm$0.02648 | 0.26 | 1.42 |
| 0.250 | / | 1.79424$\pm$0.02653 | N/A | N/A |
| 0.300 | / | 1.83215$\pm$0.03908 | 0.32 | 1.58 |
| 0.400 | / | 1.92868$\pm$0.04562 | 0.37 | 1.75 |
| 0.500 | / | 1.95660$\pm$0.04644 | 0.41 | 1.93 |
| 0.600 | / | 2.00549$\pm$0.08831 | 0.45 | 2.11 |

**References:**

47 Kresse, G. & Furthmüller, J. Efficiency of ab-initio total energy calculations for metals and semiconductors using a plane-wave basis set. *Computational Materials Science* **6**, 15–50 (1996).

48 Kresse, G. & Furthmüller, J. Efficient iterative schemes for ab initio total-energy calculations using a plane-wave basis set. *Physical Review B* **54**, 11169–11186 (1996).

49 Sun, J., Ruzsinszky, A. & Perdew, J. P. Strongly Constrained and Appropriately Normed Semilocal Density Functional. *Physical Review Letters* **115**, 036402 (2015).

50 Kitagawa, K. *et al.* A spin–orbital-entangled quantum liquid on a honeycomb lattice. *Nature* **554**, 341–345 (2018).

51 Fisher, R. A. *et al.* Electron, spin-wave, hyperfine, and phonon contributions to the low-temperature specific heat of La_0.65_Ca_0.35_MnO_3_: Effects of magnetic fields and ^16^O/^18^O exchange. *Physical Review B* **64**, 134425 (2001).

52 Hewson, A. C. *The Kondo problem to heavy fermions*. (Cambridge university press, 1997).

53 Kontani, H. Generalized Kadowaki–Woods relation in heavy fermion systems with orbital degeneracy. *Journal of the Physical Society of Japan* **73**, 515–518 (2004).

54 Kadowaki, K. & Woods, S. B. Universal relationship of the resistivity and specific heat in heavy-Fermion compounds. *Solid State Communications* **58**, 507–509 (1986).

55 Tsujii, N., Kontani, H. & Yoshimura, K. Universality in Heavy Fermion Systems with General Degeneracy. *Physical Review Letters* **94**, 057201 (2005).

56 Tsujii, N., Yoshimura, K. & Kosuge, K. Deviation from the Kadowaki–Woods relation in Yb-based intermediate-valence systems. *Journal of Physics: Condensed Matter* **15**, 1993 (2003).

57 Michor, H. *et al.* Crystal structure and Kondo lattice behavior of CeNi_9_Si. *Physical Review B* **67**, 224428 (2003).

58 Naoya, T. & Masayasu, I. The ferromagnetic Kondo-lattice compound SmFe_4_P_12_. *Journal of Physics: Condensed Matter* **15**, L229 (2003).

59 Sanada, S. *et al.* Exotic Heavy-Fermion State in Filled Skutterudite SmOs_4_Sb_12_. *Journal of the Physical Society of Japan* **74**, 246–249 (2005).

60 Kondo, J. Resistance Minimum in Dilute Magnetic Alloys. *Progress of Theoretical Physics* **32**, 37–49 (1964).

61 Hamann, D. R. New Solution for Exchange Scattering in Dilute Alloys. *Physical Review* **158**, 570–580 (1967).

62 Steglich, F. *et al.* Evidence of a Kondo Destroying Quantum Critical Point in YbRh_2_Si_2_. *Journal of the Physical Society of Japan* **83**, 061001 (2014).

63 Ernst, S. *et al.* Emerging local Kondo screening and spatial coherence in the heavy-fermion metal YbRh_2_Si_2_. *Nature* **474**, 362–366 (2011).

64 Si, Q. Global magnetic phase diagram and local quantum criticality in heavy fermion metals. *Physica B: Condensed Matter* **378-380**, 23–27 (2006).

65 Si, Q. Quantum criticality and global phase diagram of magnetic heavy fermions. *physica status solidi (b)* **247**, 476–484 (2010).
